# stochprofML: stochastic profiling using maximum likelihood estimation in R

**DOI:** 10.1186/s12859-021-03970-7

**Published:** 2021-03-15

**Authors:** Lisa Amrhein, Christiane Fuchs

**Affiliations:** 1 Institute of Computational Biology, Helmholtz Zentrum München, Ingolstädter Landstrasse 1, 85764 Neuherberg, Germany; 2 Department of Mathematics, Technical University Munich, Boltzmannstrasse 3, 85748 Garching, Germany; 3 Faculty of Business Administration and Economics, Bielefeld University, Universitätsstrasse 25, 33615 Bielefeld, Germany

**Keywords:** StochprofML, Stochastic profiling, Gene expression, Cell-to-cell heterogeneity, Mixture models, Deconvolution, Maximum likelihood estimation, R

## Abstract

**Background:**

Tissues are often heterogeneous in their single-cell molecular expression, and this can govern the regulation of cell fate. For the understanding of development and disease, it is important to quantify heterogeneity in a given tissue.

**Results:**

We present the R package stochprofML which uses the maximum likelihood principle to parameterize heterogeneity from the cumulative expression of small random pools of cells. We evaluate the algorithm’s performance in simulation studies and present further application opportunities.

**Conclusion:**

Stochastic profiling outweighs the necessary demixing of mixed samples with a saving in experimental cost and effort and less measurement error. It offers possibilities for parameterizing heterogeneity, estimating underlying pool compositions and detecting differences between cell populations between samples.

**Supplementary information:**

The online version contains supplementary material available at 10.1186/s12859-021-03970-7.

## Background

Tissues are built of cells which contain their genetic information on DNA strings, so-called *genes*. These genes can lead to the generation of *messenger RNA (mRNA)* which transports the genetic information and induces the production of *proteins*. Such mRNA molecules and proteins are modes of expression by which a cell reflects the presence, kind and activity of its genes. In this paper, we consider such *gene expression* in terms of quantities of mRNA molecules.

Gene expression is stochastic. It can differ significantly between, e.g., types of cells or tissues, and between individuals. In that case, one refers to *differential gene expression*. In particular, cells can be differentially expressed between healthy and sick tissue samples from the same origin. Moreover, cells can differ even within a small tissue sample, e.g. within a tumour that consists of several mutated cell populations. Mathematically, we regard two populations to be different if their mRNA counts follow different probability distributions. If there is more than one population in a tissue, we call it heterogeneous. The expression of such tissues can be described by mixture models. Detecting and parameterizing heterogeneities is of utmost importance for understanding development and disease.

The amount of mRNA molecules of a gene in a tissue sample can be assessed by various techniques such as microarray measurements [1, 2] or sequencing [3, 4]. Bulk measurements are suitable for analyses like mean comparisons but make it difficult to describe in-bulk heterogeneity. To infer partial information about cell populations, bulk deconvolution methods like CIBERSORT [5] require the availability of so-called signature matrices. Measurements of single cells yield the highest possible resolution. They are best suited for identification and description of heterogeneity in large and error-free datasets. In practice, however, single-cell data often comes along with high cost, effort and technical noise [6]. Heterogeneity can still be revealed given sufficient sample size and additional information such as the expression of cell cycle genes [e.g. 7]. In our work, we consider the case of comparatively small samples without further prior knowledge. Instead of considering single-cell data, we analyze the cumulative gene expression of small pools of randomly selected cells [8]. The pool size should be large enough to substantially reduce measurement error and cost, and at the same time small enough such that heterogeneity is still identifiable. The analysis of such small cell pools could add additional information that is lost in single-cell measurements due to the stress in which the cells find themselves once they are separated from their tissue.

Such new kind of data requires new analysis tools. We thus developed the algorithm stochprofML to infer single-cell regulatory states from small pools of cells [9]. In contrast to previously existing deconvolution methods, which were not tailored to small cell pools, it neither requires a priori knowledge about the mixing weights such as the csSAM [10] or DSection [11] algorithms nor about expression profiles which is required when using for example the qproq [12] or lsfit [13] tools. Only the CAM method [14] performs unsupervised deconvolution for clusters of genes, however with the aim to find marker genes. Several of these methods are implemented in the R package CellMix [15], but for the above reasons, they are not directly comparable. In [9], we still demonstrated on synthetic data how stochastic profiling led to more accurate estimates than competing approaches.

Recently, tools were developed with the aim to deconvolute bulk measurements using the available huge datasets of single-cell data or purified bulk samples such as AutoGeneS [16], dtangle [17] or CPM [18]. However, deconvolution without any basis such as purified expression datasets of subpopulations or other prior knowledge is much harder. Here we present the stochastic profiling algorithm that blindly deconvolves the joint measurements purely by applying a combinatorial mixture model.

In [9], we applied stochprofML to measurements from human breast epithelial cells and revealed the functional relevance of the heterogeneous expression of a particular gene. Fluorescence in situ hybridization confirmed that the computationally identified population fractions corresponded to experimentally detected transcriptional populations. In a second study, we applied the algorithm to clonal tumor spheroids of colorectal cancer [19]. There, a single tumor cell was cultured, and after several rounds of replication, each resulting spheroid was imaged and sequenced. However, pool sizes differed between tissue samples as each spheroid contained a different number of cells ranging from less than ten to nearly 200 cells. Therefore, we extended stochprofML to be able to handle pools of different sizes.

In this work, we present such modeling extensions alongside numerical and computational detail. We explore the performance of the algorithm in simulation studies for various settings, especially in the realistic case of uncertainty about the pool size. To expand the range of applications, we propose a test for significant differences between the estimated populations and inference of original pool compositions.

## Implementation

The stochprofML algorithm aims at maximum likelihood estimation of the corresponding model parameters. Hence, we derive the likelihood functions of the parameters and show details of the estimation and its implementation. The new elements of the most recent version of the algorithm are introduced along the line. Note that we will use a combinatorial mixture since this we aim for a blind convolution model that does not need any prior input information on the contained subpopulations or their fractions.

### Notation

**Fig. 1:**
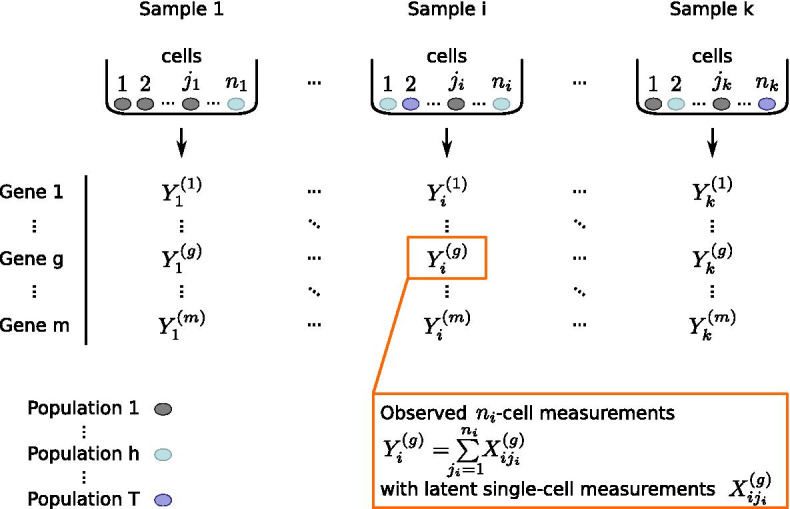
Experimental design of pooling cells into samples, measuring the pooled gene expression across several genes for which identical population structures are assumed. The table illustrates the index notation of (tissue) samples, single cells, populations and genes as well as observed and latent measurements

Suppose there are *k* (tissue) samples, indexed by $i \in \{ 1,\ldots ,k\}$. From each tissue sample *i*, we collect a pool of a known number of cells. The cells are either indexed by $j \in \{ 1,\ldots ,n\}$ if the cell pool size is the same in all measurements, or, as possible in the latest implementation, by $j_i \in \{ 1,\ldots ,n_i\}$ in case cell pool sizes vary between measurements. In the latter, more general case, the cell numbers are variable over the *k* cell pools and summarized by $\vec{n} = (n_1,\ldots , n_k )$. From each sample, the gene expression of *m* genes is measured, indexed by $g \in \{ 1,\ldots ,m\}$. We assume that each cell stems from one out of *T* cell populations, indexed by $h \in \{ 1,\ldots ,T\}$. If $T>1$ in the set of all cells of interest, the tissue is called heterogeneous. The notation is illustrated in Fig. 1. Biologically, the different cell populations correspond to different regulatory states or—especially in the context of cancer—to different (sub-)clones. For example, there might be two populations within a considered tissue: one occupying a basal regulatory state, where the expression of genes is at a low level, and one from a second regulatory state, where genes are expressed at a higher level.

### Single-cell models of heterogeneous gene expression

As described above, there are various technologies to measure gene expression. Microarrays (as considered in previous applications of stochastic profiling, see [8, 9]) measure relative gene expression, which is appropriately described in terms of continuous probability distributions. Sequencing experiments produce discrete molecule counts. However, if these numbers are large, or if preprocessing blurs the discrete character of the data, one often describes such sequencing output by continuous probability distributions as well. Conditioned on the cell population, stochprofML provides two continuous choices for the single-cell distribution of the expression of one gene:

#### Lognormal distribution

The two parameters defining a univariate lognormal distribution ${\mathcal{L}}{\mathcal{N}}(\mu ,\sigma ^2)$ are called log-mean $\mu \in {\mathbb{R}}$ and log-standard deviation $\sigma >0$. These are the mean and the standard deviation of the normally distributed random variable $\log (X)$, the natural logarithm of *X*. The probability density function (PDF) of *X* is given by

$$\begin{aligned} f_\text{LN}(x|\mu , \sigma ^2) = \frac{1}{\sqrt{2\pi }\sigma x}\,\exp \left( -\frac{(\log x-\mu )^2}{2\sigma ^2}\right) \quad \text{for }x>0. \end{aligned}$$

A random variable $X\sim {\mathcal{L}}{\mathcal{N}}(\mu ,\sigma ^2)$ has expectation and variance

1 $$\begin{aligned} \text{E}(X)= \exp \left( \mu +\frac{\sigma ^2}{2}\right) \qquad \text{and}\qquad \text{Var}(X)=\exp \left( 2\mu +\sigma ^2\right) \left( \exp \left( \sigma ^2\right) -1\right). \end{aligned}$$

#### Exponential distribution

An exponential distribution ${\mathcal{E}}\! {\mathcal{X}}\! {\mathcal{P}}(\lambda )$ is defined by the rate parameter $\lambda >0$. The PDF is given by

$$\begin{aligned} f_{E\! X\! P}(x| \lambda ) = \lambda \exp \left( -\lambda x \right) \quad \text{for }x \ge 0. \end{aligned}$$

A random variable $X\sim {\mathcal{E}}\! {\mathcal{X}}\! {\mathcal{P}}(\lambda )$ has expectation and variance

$$\begin{aligned} \text{E}(X)= \frac{1}{\lambda }\qquad \text{and}\quad \text{Var}(X)= \frac{1}{\lambda ^2}. \end{aligned}$$

In general, the lognormal distribution is an appropriate description of continuous gene expression [20]. With its two parameters, it is more flexible than the exponential distribution. However, the lognormal distribution cannot model zero gene expression as often present in real-world applications. In case of zeros in the data, it could be modified by adding small values such as 0.0001, or one uses the exponential distribution to model this kind of expression. This distribution is an obvious choice to model zero and very low expression as its support includes zero, and with only one distribution parameter it avoids unnecessary model complexity. Furthermore, we will show later that the exponential distribution can be convoluted in closed form.

In case of *T* cell populations, we describe the expression of one gene by a stochastic mixture model. Let $\left( p_1, \ldots , p_T\right)$ with $p_1+\cdots +p_T=1$ denote the fractions of populations in the overall set of cells. stochprofML offers the following three mixture models:

#### Lognormal–lognormal (LN–LN)

Each population *h* is represented by a lognormal distribution with population-specific parameter $\mu _h$ (different for each population *h*) and identical $\sigma$ for all *T* populations. The single-cell expression *X* that originates from such a mixture of populations then follows

$$\begin{aligned} X \sim {\left\{ \begin{array}{ll} {\mathcal{L}}{\mathcal{N}}(\mu _1,\sigma ^2) &{} \text{with probability } p_1\\ \vdots &{}\\ {\mathcal{L}}{\mathcal{N}}(\mu _h,\sigma ^2) &{} \text{with probability } p_h\\ \vdots &{}\\ {\mathcal{L}}{\mathcal{N}}(\mu _T,\sigma ^2) &{} \text{with probability } \left( 1-\sum _{h=1}^{T-1} p_h\right) . \end{array}\right. } \end{aligned}$$

#### Relaxed lognormal–lognormal (rLN–LN)

This model is similar to the LN–LN model, but each population *h* is represented by a lognormal distribution with a different parameter set ($\mu _h$, $\sigma _h$). The single-cell expression *X* follows

$$\begin{aligned} X \sim {\left\{ \begin{array}{ll} {\mathcal{L}}{\mathcal{N}}(\mu _1,\sigma _1^2) &{} \text{with probability } p_1\\ \vdots &{}\\ {\mathcal{L}}{\mathcal{N}}(\mu _h,\sigma _h^2)&{} \text{with probability } p_h\\ \vdots &{}\\ {\mathcal{L}}{\mathcal{N}}(\mu _T,\sigma _T^2) &{} \text{with probability } \left( 1-\sum _{h=1}^{T-1} p_h\right) . \end{array}\right. } \end{aligned}$$

#### Exponential–lognormal (EXP–LN)

Here, one population is represented by an exponential distribution with parameter $\lambda$, and all remaining $T-1$ populations are modeled by lognormal distributions analogously to LN–LN, i.e. with population-specific parameters $\mu _h$ and identical $\sigma$. The single-cell expression *X* then follows

$$\begin{aligned} X \sim {\left\{ \begin{array}{ll} {\mathcal{L}}{\mathcal{N}}(\mu _1,\sigma ^2)&{} \text{with probability } p_{1}\\ \vdots &{}\\ {\mathcal{L}}{\mathcal{N}}(\mu _h,\sigma ^2)&{} \text{with probability } p_{h}\\ \vdots &{}\\ {\mathcal{L}}{\mathcal{N}}(\mu _{T-1},\sigma ^2) &{} \text{with probability } p_{T-1} \\ {\mathcal{E}}\! {\mathcal{X}}\! {\mathcal{P}}(\lambda ) &{} \text{with probability } \left( 1-\sum _{h=1}^{T-1} p_h\right) .\\ \end{array}\right. } \end{aligned}$$

The LN–LN model is a special case of the rLN–LN model. It assumes identical $\sigma$ across all populations. Biologically, this assumption is motivated by the fact that, for the lognormal distribution, identical $\sigma$ lead to identical coefficient of variation

$$\begin{aligned} \text{CV}(X) = \frac{\sqrt{\text{Var}(X)}}{\text{E}(X)}= \sqrt{\exp (\sigma ^2)-1} \end{aligned}$$

even for different values of $\mu$. In other words, the linear relationship between the mean expression and the standard deviation is maintained across cell populations in the LN–LN model. The appropriateness of the different mixture models can be discussed both biologically and in terms of statistical model choice.

Within one set of genes under consideration, we assume that the same type of model (LN–LN, rLN–LN, EXP–LN) is appropriate for all genes. The parameter values, however, may differ. In case of *T* cell populations, we describe the single-cell gene expression $X^{(g)}$ for gene *g* by a mixture distribution with PDF

$$\begin{aligned}{}&f_\text{T-pop}\left( x^{(g)} | \right. \, \left. \varvec{\theta }^{(g)}, \varvec{p}\right) \\&\quad =p_1 f_1\left( x^{(g)}|\theta _1^{(g)}\right) + \cdots + p_h f_h\left( x^{(g)}|\theta _h^{(g)}\right) +\cdots + \left( 1-\sum _{h=1}^{T-1} p_h\right) f_T\left( x^{(g)}|\theta _T^{(g)}\right) , \end{aligned}$$

where $f_h$ with $h\in \{1,\ldots , T\}$ represents the PDF of population *h* that can be either lognormal or exponential, and $\varvec{\theta }^{(g)}=\{\theta _1^{(g)},\ldots ,\theta _T^{(g)}\}$ are the (not necessarily disjoint) distribution parameters of the *T* populations for gene *g*.

#### Example: Mixture of two populations—Part 1

We exemplify the two-population case. Here, the PDF of the mixture distribution for gene *g* reads

$$\begin{aligned} f_\text{2-pop} (x^{(g)} |\varvec{\theta }^{(g)})= p f_1 (x^{(g)} |\theta _1^{(g)})+(1-p)f_2 (x^{(g)} |\theta _2^{(g)}), \end{aligned}$$

where *p* is the probability of the first population. The univariate distributions $f_1^{(g)}$ and $f_2^{(g)}$ depend on the chosen model:

**LN–LN:** $f_1 (x^{(g)} |\theta _1^{(g)}) = f_\text{LN}(x^{(g)} | \mu _1^{(g)}, \sigma ^2)$ and $f_2(x^{(g)} |\theta _2^{(g)}) = f_\text{LN}(x^{(g)} | \mu _2^{(g)}, \sigma ^2)$, i.e. there are four unknown parameters: $p, \mu _1^{(g)}, \mu _2^{(g)}$ and $\sigma ^2$.

**rLN–LN:** $f_1 (x^{(g)} |\theta _1^{(g)}) = f_\text{LN}(x^{(g)} | \mu _1^{(g)}, {\sigma _1}^2)$ and $f_2(x^{(g)} |\theta _2^{(g)}) = f_\text{LN}(x^{(g)} | \mu _2^{(g)}, {\sigma _2}^2)$ i.e. there are five unknown parameters: $p, \mu _1^{(g)}, \mu _2^{(g)}, {\sigma _1}^2$ and ${\sigma _2}^2$.

**EXP–LN:** $f_1(x^{(g)} |\theta _1^{(g)}) = f_\text{LN}(x^{(g)} | \mu ^{(g)}, {\sigma }^2)$ and $f_2(x^{(g)} |\theta _2^{(g)}) = f_\text{EXP}(x^{(g)} | \lambda ^{(g)})$. i.e. there are four unknown parameters: $p, \mu ^{(g)}$, $\sigma ^2$ and $\lambda ^{(g)}$. Note that although each lognormal population has its individual $\sigma$, these $\sigma$-values remain identical across genes in all models.

### Small-pool models of heterogeneous gene expression

**Fig. 2:**
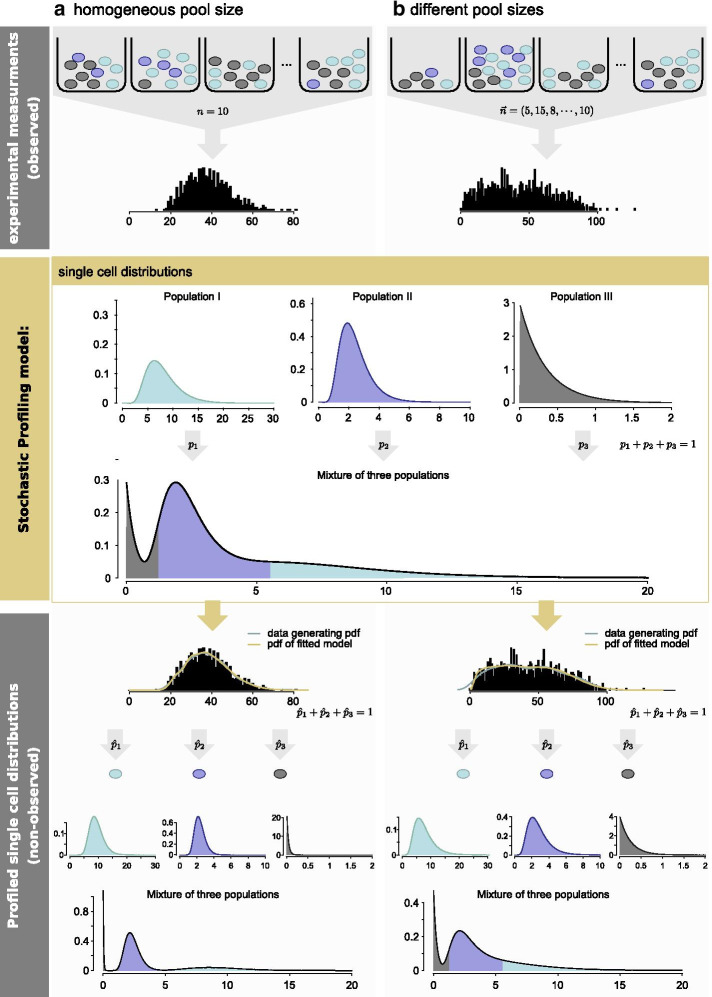
Stochastic Profiling can be performed either on measurements of **a** homogeneous pool size of $n$ cells or of **b** different pool sizes given by the cell number vector $\vec{n}{}$. In both cases, the stochprofML algorithm estimates the parameters for the specified number of populations from pooled data, leading to inferred single-cell distributions for each population. Additional File 2 describes how this density is visualized here

stochprofML is tailored to analyze gene expression measurements of small pools of cells, beyond the analysis of standard single-cell gene expression data. In other words, the single-cell gene expression $X_{ij_i}^{(g)}$ described above is assumed latent. Instead, we consider observations

2 $$\begin{aligned} Y_i^{(g)} = \sum _{j_i=1}^{n_i} X_{ij_i}^{(g)} \end{aligned}$$

for $i=1,\ldots ,k$, which represent the overall gene expression of the *i*th cell pool for gene *g*. In the first version of stochprofML, pools had to be of equal size *n*, i.e. for each measurement $Y_{i}^{(g)}$ one had to extract the same number of cells from each tissue sample. This was a restrictive assumption from the experimental point of view. The recent extension of stochprofML allows each cell pool *i* to contain a different number $n_i$ of cells (see also Figs. 1 and 2).

The algorithm aims to estimate the single-cell population parameters despite the fact that measurements are available only in convoluted form. To that end, we derive the likelihood function of the parameters in the convolution model (2), where we assume the gene expression of the single cells to be independent within a tissue sample. For better readability, we suppress for now the superscript (*g*) and introduce it again later.

The derivation of the distribution of $Y_i$ is described in Additional file 1. The corresponding PDF $f_{n_i}(y_i|\varvec{\theta },\varvec{p})$ of an observation $y_i$ which represents the overall gene expression from sample *i* (consisting of $n_i$ cells) is given by

3 $$\begin{aligned}{}&f_{n_i} \left( y_i| \! \right. \, \left. \varvec{\theta }, \varvec{p}\right) = \nonumber \\&\quad \sum _{\ell _1=0}^{n_i}\sum _{\ell _2=0}^{{n_i}-\ell _1} \cdots \sum _{\ell _{T-1}=0}^{{n_i}-\sum _{h=1}^{T-2} \ell _h} {{n_i} \atopwithdelims (){\ell _1,\ell _2,\ldots ,\ell _T}}p_1^{\ell _1}p_2^{\ell _2}\cdots p_T^{\ell _T} f_{ ( \ell _1,\ell _2,\ldots ,\ell _T)}\left( y_i|\varvec{\theta } \right) , \end{aligned}$$

where $\ell _T = n_i-\sum _{h = 1}^{T-1} \ell _h$ and $p_T = 1-\sum _{h = 1}^{T-1} p_h$. Here, $f_{(\ell _1,\ell _2,\ldots ,\ell _T)}$ describes the PDF of a pool of $n_i$ cells with *known* composition of the single populations, i.e. it is known that there are $\ell _1$ cells from population 1, $\ell _2$ cells from population 2 etc. ${n_i \atopwithdelims (){\ell _1,\ell _2,\ldots ,\ell _T} } p_1^{\ell _1}p_2^{\ell _2}\cdots p_T^{\ell _T}$ represents the multinomial probability of obtaining exactly this composition $(\ell _1,\ldots ,\ell _T)$ using the multinomial coefficient ${n_i \atopwithdelims (){\ell _1,\ell _2,\ldots ,\ell _T} }=n_i!/(\ell _1! \ldots \ell _T!)$. Equation (3) sums up over all possible compositions $(\ell _1,\ldots ,\ell _T)$ with $\ell _1,\ldots ,\ell _T\in {\mathbb{N}}_0$ and $\ell _1+\cdots +\ell _T=n_i$. Taken together, $f_{n_i}(y_i|\varvec{\theta }, \varvec{p})$ determines the PDF of $y_i$ with respect to each possible combination of $n_i$ cells of *T* populations.

Thus, the calculation of $f_{n_i}(y_i|\varvec{\theta }, \varvec{p})$ requires knowledge of $f_{(\ell _1,\ell _2,\ldots ,\ell _T)}(y_i|\varvec{\theta })$ . The derivation of this PDF depends on the choice of the single-cell model (LN–LN, rLN–LN, or EXP–LN) that was made for $X_{ij_i}$ .

#### LN–LN

$$\begin{aligned} f_{(\ell _1,\ldots ,\ell _h,\ldots ,\ell _T)}(y_i|\varvec{\theta }) = f^\text{LN-LN}_{(\ell _1,\ldots ,\ell _h,\ldots ,\ell _T)} (y_i|\mu _1,\ldots ,\mu _h,\ldots , \mu _T,\sigma ^2) \end{aligned}$$

is the density of a sum $Y_i=X_{i1}+\cdots +X_{in_i}$ of ${n_i}$ independent random variables with

$$\begin{aligned} X_{ij_i} \sim {\left\{ \begin{array}{ll} {\mathcal{L}}{\mathcal{N}}(\mu _1,\sigma ^2) &{} \text{if } 1\le j_i\le J_1\\ \vdots &{}\\ {\mathcal{L}}{\mathcal{N}}(\mu _h,\sigma ^2) &{} \text{if } J_{h-1}< j_i\le J_h\\ \vdots &{}\\ {\mathcal{L}}{\mathcal{N}}(\mu _T,\sigma ^2) &{} \text{if } J_{T-1}< j_i\le J_T=n_i, \end{array}\right. } \end{aligned}$$

with $J_1 = \ell _1,\ldots , J_h = \ell _1+\ell _2 + \cdots + \ell _h, \ldots , J_T = \ell _1 + \ell _2 + \cdots + \ell _T = n_i$. $Y_i$ is the convolution of random variables $X_{i1},\ldots ,X_{in_i}$, which is here the convolution of *T* sub-convolutions: a convolution of $\ell _1$ times ${\mathcal{L}}{\mathcal{N}}(\mu _1,\sigma ^2)$, plus a convolution of $\ell _2$ times ${\mathcal{L}}{\mathcal{N}}(\mu _2,\sigma ^2)$, and so on, up to a convolution of $\ell _T$ times ${\mathcal{L}}{\mathcal{N}}(\mu _T,\sigma ^2)$.

There is no analytically explicit form for the convolution of lognormal random variables. Hence, $f^\text{LN-LN}_{(\ell _1,\ldots ,\ell _h,\ldots ,\ell _T)}$ is approximated using the method by [21]. That is, the distribution of the sum $A_1+\cdots +A_m$ of independent random variables

$A_i\sim {\mathcal{L}}{\mathcal{N}}(\mu _{A_i},\sigma _{A_i}^2)$ is approximated by the distribution of a random variable $B\sim {\mathcal{L}}{\mathcal{N}}(\mu _B,\sigma _B^2)$ such that

$$\begin{aligned} \text{E}(B)=\text{E}(A_1+\cdots +A_m) \quad \text{and}\quad \text{Var}(B)=\text{Var}(A_1+\cdots +A_m). \end{aligned}$$

According to Eq. (1), that means that $\mu _B$ and $\sigma _B$ are chosen such that the following equations are fulfilled:

$$\begin{aligned} \exp \left( \mu _B+\frac{\sigma _B^2}{2}\right) = \exp \left( \mu _{A_1}+\frac{\sigma _{A_1}^2}{2}\right) + \cdots + \exp \left( \mu _{A_m}+\frac{\sigma _{A_m}^2}{2}\right) =:\Gamma \end{aligned}$$

and

$$\begin{aligned}{}&\exp \!\left( 2\mu _B+\sigma _B^2\right) \! \left( \exp \left( \sigma _B^2\right) \!-\!1\right) = \\&\quad \exp \left( 2\mu _{A_1}+\sigma _{A_1}^2\right) \!\left( \exp \left( \sigma _{A_1}^2\right) \!-\!1\right) + \cdots + \exp \left( 2\mu _{A_m}+\sigma _{A_m}^2\right) \!\left( \exp \left( \sigma _{A_m}^2\right) -1\right) =:\Delta . \end{aligned}$$

That is achieved by choosing

$$\begin{aligned} \mu _B = \log (\Gamma )-\frac{1}{2} \sigma _B^2 \quad \text{and}\quad \sigma _B^2 = \log \left( \frac{\Delta }{\Gamma ^2}+1\right) . \end{aligned}$$

This approximation is implemented in the function d.sum.of.lognormals(). The overall PDF is computed through d.sum.of.mixtures.LNLN().

#### rLN–LN

$$\begin{aligned} f_{(\ell _1,\ldots ,\ell _h,\ldots ,\ell _T)}(y_i|\varvec{\theta }) = f^\text{rLN-LN}_{(\ell _1,\ldots ,\ell _h,\ldots ,\ell _T)}(y_i|\mu _1,\ldots ,\mu _h,\ldots , \mu _T,\sigma _1^2,\ldots ,\sigma _h^2,\ldots ,\sigma _T^2) \end{aligned}$$

is the PDF of a sum $Y_i=X_{i1}+\cdots +X_{in_i}$ of ${n_i}$ independent random variables with

$$\begin{aligned} X_{ij_i} \sim {\left\{ \begin{array}{ll} {\mathcal{L}}{\mathcal{N}}(\mu _1,\sigma _1^2) &{} \text{if } 1\le j_i\le J_1\\ \vdots &{}\\ {\mathcal{L}}{\mathcal{N}}(\mu _h,\sigma _h^2) &{} \text{if } J_{h-1}< j_i\le J_h\\ \vdots &{}\\ {\mathcal{L}}{\mathcal{N}}(\mu _T,\sigma _T^2) &{} \text{if } J_{T-1}< j_i\le J_T=n_i, \end{array}\right. } \end{aligned}$$

with $J_1 = \ell _1,\ldots , J_h = \ell _1+\ell _2 + \cdots + \ell _h, \ldots , J_T = \ell _1 + \cdots + \ell _T = n_i$. Again, $f^\text{rLN-LN}_{(\ell _1,\ldots ,\ell _h,\ldots ,\ell _T)}$ is approximated using the method by [21], analogously to the LN–LN model. It is implemented in d.sum.of.mixtures.rLNLN().

#### EXP–LN

$$\begin{aligned} f_{(\ell _1,\ell _2,\ldots ,\ell _T)}(y_i|\varvec{\theta }) =f^\text{EXP-LN}_{(\ell _1,\ell _2,\ldots ,\ell _T)}(y_i|\lambda ,\mu _1,\ldots , \mu _{T-1},\sigma ^2) \end{aligned}$$

is the density of a sum $Y_i=X_{i1}+\cdots +X_{in_i}$ of $n_i$ independent random variables with

$$\begin{aligned} X_{ij_i} \sim {\left\{ \begin{array}{ll} {\mathcal{L}}{\mathcal{N}}(\mu _1,\sigma ^2) &{} \text{if } 1\le j_i\le J_1\\ \vdots &{}\\ {\mathcal{L}}{\mathcal{N}}(\mu _h,\sigma ^2) &{} \text{if } J_{h-1}< j_i\le J_h\\ \vdots &{}\\ {\mathcal{L}}{\mathcal{N}}(\mu _{T-1},\sigma ^2) &{} \text{if } J_{T-2}< j_i\le J_{T-1}\\ {\mathcal{E}}\! {\mathcal{X}}\! {\mathcal{P}}(\lambda ) &{} \text{if } J_{T-1}< j_i\le J_T=n_i, \end{array}\right. } \end{aligned}$$

with $J_1 = \ell _1,\ldots , J_h = \ell _1+\ell _2 + \cdots + \ell _h, \ldots , J_T = \ell _1 + \cdots + \ell _T = n_i$. The sum of independent exponentially distributed random variables with equal intensity parameter follows an Erlang distribution [22], which is a gamma distribution with integer-valued shape parameter that represents the number of exponentially distributed summands. Thus, the PDF for the EXP–LN mixture model is the convolution of one Erlang (or gamma) distribution (namely the sum of all exponentially distributed summands) and one lognormal distribution [namely the sum of all lognormally distributed summands, again using the approximation method by 21]. The PDF for this convolution is not known in analytically explicit form but expressed in terms of an integral that is solved numerically through the function lognormal.exp.convolution(). Its computation thus takes substantially longer in terms of run time than for LN–LN. The overall PDF of the EXP–LN model is implemented in d.sum.of.mixtures.EXPLN().

#### Example: Mixture of two populations—Part 2

In this example of the two-population model, let each observation consist of the same number of $n=10$ cells. Then $Y_i$ is a 10-fold convolution, and the PDF (3) simplifies to

4 $$\begin{aligned} f_{10}\left( y_i|\varvec{\theta },\varvec{p} \right) =\sum _{\ell =0}^{10} {10 \atopwithdelims ()\ell } p^\ell (1-p)^{10-\ell } f_{(\ell ,10-\ell )}\left( y_i | \varvec{\theta } \right) , \end{aligned}$$

where $f_{(\ell ,10-\ell )}$ is the PDF of the sum $Y_i$ of ten independent random variables, that is

$Y_i=X_{i1}+\cdots +X_{i 10}$. This PDF depends on the particular chosen model:

#### LN–LN

$$\begin{aligned} f_{(\ell ,10-\ell )}(y_i|\varvec{\theta })=f^\text{LN-LN}_{(\ell ,10-\ell )} (y_i|\mu _1,\mu _2,\sigma ^2) \end{aligned}$$

is the PDF of a sum $Y_i=X_{i1}+\cdots +X_{i 10}$ of ten independent random variables with

$$\begin{aligned} X_{ij} \sim {\left\{ \begin{array}{ll} {\mathcal{L}}{\mathcal{N}}(\mu _1,\sigma ^2) &{} \text{if } 1\le j \le \ell \\ {\mathcal{L}}{\mathcal{N}}(\mu _2,\sigma ^2) &{} \text{if } \ell < j\le 10. \end{array}\right. } \end{aligned}$$

#### rLN–LN

$$\begin{aligned} f_{(\ell ,10-\ell )}(y_i|\varvec{\theta })=f^\text{rLN-LN}_{(\ell ,10-\ell )} (y_i|\mu _1,\mu _2,\sigma _1^2, \sigma _2^2) \end{aligned}$$

is the PDF of a sum $Y_i=X_{i1}+\cdots +X_{i 10}$ of ten independent random variables with

$$\begin{aligned} X_{ij} \sim {\left\{ \begin{array}{ll} {\mathcal{L}}{\mathcal{N}}(\mu _1,\sigma _1^2) &{} \text{if } 1\le j \le \ell \\ {\mathcal{L}}{\mathcal{N}}(\mu _2,\sigma _2^2) &{} \text{if } \ell < j\le 10. \end{array}\right. } \end{aligned}$$

#### EXP–LN

$$\begin{aligned} f_{(\ell ,10-\ell )}(y_i|\varvec{\theta })= f^\text{EXP-LN}_{(\ell ,10-\ell )}(y_i|\lambda ,\mu ,\sigma ^2) \end{aligned}$$

is the PDF of a sum $Y_i=X_{i1}+\cdots +X_{i 10}$ of ten independent random variables with

$$\begin{aligned} X_{ij} \sim {\left\{ \begin{array}{ll} {\mathcal{L}}{\mathcal{N}}(\mu ,\sigma ^2) &{} \text{if } 1\le j \le \ell \\ {\mathcal{E}}\! {\mathcal{X}}\! {\mathcal{P}}(\lambda ) &{} \text{if } \ell < j\le 10.\\ \end{array}\right. } \end{aligned}$$

### Likelihood function

Overall, after re-introducing the superscript (*g*) for measurements of genes $g=1,\ldots ,m$, we obtain the PDF

5 $$\begin{aligned}{}&f_{n_i}\left( y_i^{(g)}| \right. \left. \varvec{\theta }^{(g)}, \varvec{p}\right) = \nonumber \\&\quad \sum _{\ell _1=0}^{n_i}\sum _{\ell _2=0}^{{n_i}-\ell _1}\cdots \sum _{\ell _{T-1}=0}^{{n_i}-\sum _{h=1}^{T-2} \ell _h} {{n_i} \atopwithdelims (){\ell _1,\ell _2,\ldots ,\ell _T}}p_1^{\ell _1}p_2^{\ell _2}\cdots p_T^{\ell _T} f_{ ( \ell _1,\ell _2,\ldots ,\ell _T)}\left( y_i^{(g)}|\varvec{\theta }^{(g)} \right) \end{aligned}$$

with model-specific choice of $f_{ ( \ell _1,\ell _2,\ldots ,\ell _T)}$. While $\varvec{n}=(n_1,\ldots ,n_k)$ is considered known, we aim to infer the unknown model parameters $\varvec{\theta }=\{\varvec{\theta }^{(1)}, \ldots ,\varvec{\theta }^{(m)},\varvec{p}\}$ by maximum likelihood estimation. Assuming independent observations ${\varvec{y}}=\{y_i^{(g)}|i=1,\ldots ,k;g=1,\ldots ,m\}$ of $Y_i^{(g)}$ for *m* genes and *k* tissue samples, where sample *i* contains $n_i$ cells, the likelihood function is given by

$$\begin{aligned} L(\varvec{\theta }|\varvec{y}) = \prod _{g=1}^m\prod _{i=1}^k f_{n_i}\left( y_i^{(g)}| \varvec{\theta }^{(g)}, \varvec{p}\right) . \end{aligned}$$

Consequently, the log-likelihood function of the model parameters reads

6 $$\begin{aligned} \ell (\varvec{\theta }|\varvec{y}) = \sum _{g=1}^m \sum _{i=1}^k \log \left[ f_{n_i} \left( y_i^{(g)}|\varvec{\theta }^{(g)}, \varvec{p}\right) \right] . \end{aligned}$$

#### Example: Mixture of two populations—Part 3

Returning to the two-population example with 10-cell pools, the log-likelihood for $k=100$ tissue samples and $m=5$ genes is given by

$$\begin{aligned} \ell (\varvec{\theta }|\varvec{y}) = \sum _{g=1}^5 \sum _{i=1}^{100} \log \left[ f_{10}\left( y_i^{(g)}|\varvec{\theta }^{(g)},\varvec{p} \right) \right] , \end{aligned}$$

where $f_{10}\left( y_i^{(g)}|\varvec{\theta }^{(g)},\varvec{p} \right)$ is given by Eq. (4).

### Maximum likelihood estimation

The stochprofML algorithm aims to infer the unknown model parameters using maximum likelihood estimation. As input, we expect an $m\times k$ data matrix of pooled gene expression, known cell numbers $\vec{n}$, the assumed number of populations *T* and the choice of single-cell distribution (LN–LN, rLN–LN, EXP–LN). Based on this input, the algorithm aims to find parameter values of $\varvec{\theta }=\{\varvec{\theta }^{(1)},\ldots ,\varvec{\theta }^{(m)},\varvec{p}\}$ that maximize $\ell (\varvec{\theta }|\varvec{y})$ as given by Eq. (6). This section describes practical aspects of the optimization procedure.

#### Example: Mixture of two populations—Part 4

Several challenges occur during parameter estimation. We explain these on the two-population LN–LN example: First, we aim to ensure parameter identifiability. This is achieved for the two-population LN–LN model by constraining the parameters to fulfil either $p\le 0.5$ or $\mu _1 > \mu _2$. Otherwise, the two combinations $(p,{\varvec{\mu }}_1,{\varvec{\mu }}_2,\sigma )$ and $(1-p,{\varvec{\mu }}_2,{\varvec{\mu }}_1,\sigma )$ would yield identical values of the likelihood function and could cause computational problems. For our implementation, we preferred the second possibility, i.e. $\mu _1 > \mu _2$. The alternative, i.e. requiring $p\le 0.5$, led to switchings between $\mu _1$ and $\mu _2$ in case of $p \approx 0.5$. As a second measure, we implement unconstrained rather than constrained optimization: Instead of estimating $(p,{\varvec{\mu }}_1,{\varvec{\mu }}_2,\sigma )$ under the constraints $p\in [0,1]$, $\mu _1 > \mu _2$ and $\sigma >0$, the parameters are transformed to $(\text{logit}(p),{\varvec{\mu }}_1,{\varvec{\mu }}_2,\log (\sigma ))$, and an unconstrained optimization method is used. This is substantially faster.

The aforementioned transformations are likewise employed for all other models (rLN–LN and EXP–LN) and population numbers. In particular, $\sigma$ and $\lambda$ are log-transformed, and the lognormal populations are ordered according to the log-means $\mu _h^{(1)}$ of the first gene in the gene list. The population probabilities are transformed to ${\mathbb{R}}$ such that they still sum up to one after back-transformation. For details, see Additional file 3.

The log-likelihood function is multimodal. Thus, a single application of some gradient-based optimization method does not suffice to find a global maximum. Instead, two approaches are combined which are alternately executed: First, a grid search is performed, where the log-likelihood function is computed at many randomly drawn parameter values. In the second step, the (computationally more costly) Nelder-Mead algorithm [23] is repeatedly executed at few points. This way, high likelihood regions can be identified with low computational cost. A next grid search again explores the regions around the obtained local maxima, followed by another Nelder-Mead optimization. Here, the starting values are randomly drawn from the high-likelihood regions found before. This combination of grid search and local optimization is carried out three times. The whole procedure is repeated five times by default, with the aim to find an overall optimal parameter combination, but this number can be changed using the loops parameter of the function stochprof.loop(). If until.convergence is set to TRUE, the loops will be exited as soon as the obtained improvement in the likelihood during the last round is less than $5\times 10^{-5}$.

If a dataset contains gene expressions for *m* genes, and if we assume *T* populations, there are at minimum $T(m+1)$ parameters which one seeks to estimate depending on the model framework. This is computationally difficult, because the number of modes of the log-likelihood function increases with the number of parameters. The performance of the numerical optimization crucially depends on the quality of the starting values, and a large number of restarts is required. When analyzing a large gene cluster, it is advantageous to start by considering small clusters and use the derived estimates as initial guesses for larger clusters. This is implemented in the function stochprof.loop() (parameter subgroups and demonstrated in analyze.toycluster()).

Approximate marginal 95% confidence intervals for the parameter estimates are obtained as follows: We numerically compute the Hessian matrix of the negative log-likelihood function on the unrestricted parameter space and evaluate it at the (transformed) maximum likelihood estimator. Denote by $d_i$ the *i*th diagonal element of the inverse of this matrix. Then the confidence bounds for the *i*th transformed parameter $\theta _i$ are

$$\begin{aligned} \hat{\theta }_i \pm 1.96\sqrt{d_i}. \end{aligned}$$

We obtain respective marginal confidence intervals for the original true parameters by back-transformation of the above bounds. This approximation is especially appropriate in the two-population example for the parameters *p* and $\sigma$ when conditioning on ${\varvec{\mu }}_1$ and ${\varvec{\mu }}_2$. In this case, in practice, the profile likelihood is seemingly unimodal.

Run times for maximum likelihood estimation differ substantially between two- and three-population models, and also between LN–LN, rLN–LN and EXP–LN. The latter is due to the integral convolution of an exponential and an Erlang distribution in EXP–LN as described above. Table 1 displays run times using the R function microbenchmark() on simulated data.

**Table 1:** Run times for maximum likelihood estimation for LN–LN, rLN–LN and EXP–LN models with *T* = 2 and *T* = 3 populations

*T*	LN–LN	rLN–LN	EXP–LN
2	13.00 (12.55–18.99)	27.06 (17.04–34.76)	16,762.22 (10,764.77–21,576.25)
3	96.76 (47.07–130.92)	162.59 (86.84–346.98)	148,785.38 (100,248.84–184,789.46)

The study was performed on simulated data using the R function microbenchmark(). Reported numbers are run times in seconds across five repetitions: median (min - max)

#### Example: Mixture of three populations

**Fig. 3:**
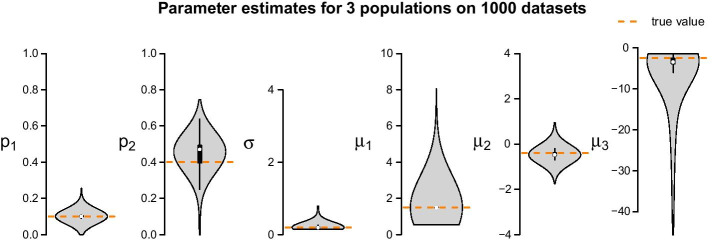
Parameter estimates for the LN–LN model on 1000 simulated 10-cell datasets. The true underlying population parameters are $p_1=0.1,\,p_2=0.4,\,\mu _1=1.5,\,\mu _2=-0.4,\,\mu _3=-2.5\,\text{and}\,\,\sigma =0.2$, as indicated by the orange dashed lines

Figure 3 shows estimation results for an LN–LN model with three populations, based on synthetic 10-cell data. (Synthetic data generation is described later in this text.) 1000 10-cell datasets each with $k = 1000$ observations were generated using underlying population parameters $p_1=0.1$, $p_2=0.4$, $\mu _1=1.5$, $\mu _2=-0.4$, $\mu _3=-2.5$ and $\sigma =0.2$.

### Model choice

By increasing the number *T* of populations, we can describe the observed data more precisely, but this comes at the cost of potential overfitting. For example, a three-population LN–LN model may lead to a larger likelihood at the maximum likelihood estimator than a two-population LN–LN model on the same dataset. However, the difference may be small, and the additional third population may not lead to a gain of knowledge. For example, the estimated population probability $\hat{p}_3$ may be tiny, or the log-means of the second and third population, ${\hat{\mu }}_{2}$ and ${\hat{\mu }}_{3}$ might hardly be distinguishable from each other.

To objectively find a trade-off between necessary complexity and sufficient interpretability, we employ the Bayesian information criterion [BIC, 24]:

$$\begin{aligned} \text{BIC}(\hat{{\varvec{\theta }}})=-2\ell (\hat{{\varvec{\theta }}})+ {\log {k}} \dim (\hat{{\varvec{\theta }}}), \end{aligned}$$

where $\hat{{\varvec{\theta }}}$ is the maximum likelihood estimate of the respective model, $\dim (\hat{{\varvec{\theta }}})$ the number of parameters and *k* the size of the dataset. From the statistics perspective, the model with smallest BIC is considered most appropriate among all considered models.

In practice, it is required to estimate all models of interest separately with the stochprofML algorithm, e.g. the LN–LN model with one, two and three populations, and/or the respective rLN–LN and EXP–LN models. The BIC values are returned by the function stochprof.loop().

## Results and discussion

This section illustrates the usage of the stochprofML package for simulation and parameter estimation. Afterwards we demonstrate the performance of the estimation depending on pool sizes, true parameter values and in case of uncertainty about pool sizes. We investigate what we can learn from the parameter estimates about the heterogeneous populations and about sample compositions. These investigations shed light on the algorithm’s performance from a statistical point of view and complement the experimental validation we performed in [9]. All scripts used in these studies can be found in our open GitHub repository https://github.com/fuchslab/Stochastic_Profiling_in_R.

### Usage of stochprofML

There are two ways to use the stochprofML package: (1) Two interactive functions stochasticProfilingData() and stochasticProfilingML() provide low-level access to synthetic data generation and maximum likelihood parameter estimation without requiring advanced programming knowledge. They guide the user through entering the relevant input parameters: Working as question-answer functions, they ask for prompting the data (or file name), the number of cells per sample, the number of genes etc. An example of the use of the interactive functions can be found in Additional file 5. (2) The direct usage of the package’s R functions allows more flexibility and is illustrated in the following.

#### Synthetic data generation

**Fig. 4:**
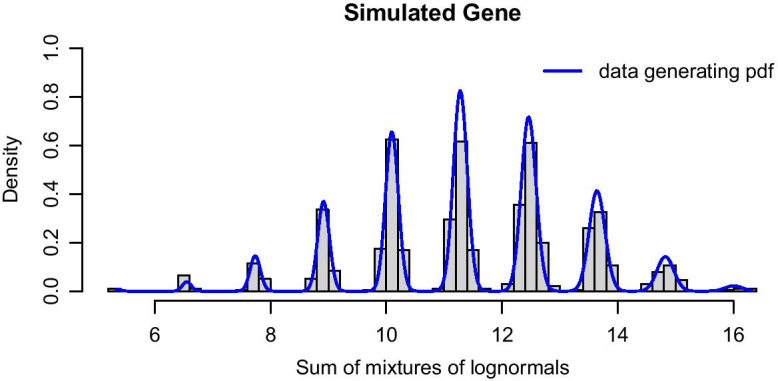
Histogram of 1000 synthetic 10-cell observations, together with theoretical PDF. We assumed a two-population LN–LN model with parameters $p=0.62,\,\mu _1=0.47,\,\mu _2=-0.87\,\text{and}\,\,\sigma =0.03$

We first generate a dataset of $k=1000$ sample observations, where each sample consists of $n=10$ cells. We choose a single-cell model with two populations, both of lognormal type, i.e. we use the LN–LN model. Let us assume that the overall population of interest is a mixture of $62\%$ of population 1 and $38\%$ of population 2, i.e. $p_1=0.62$. As population parameters we choose $\mu _1 = 0.47$, $\mu _2 = -0.87$ and $\sigma =0.03$. Synthetic gene expression data for one gene is generated as follows: 
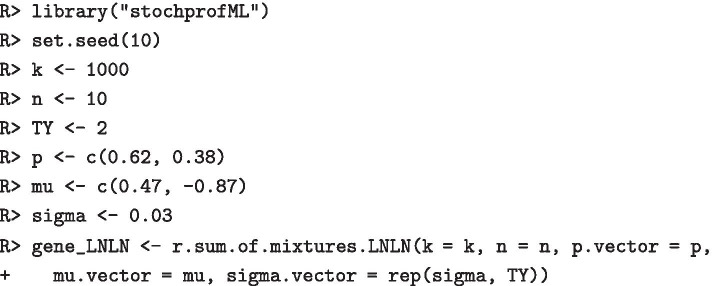


Figure 4 shows a histogram of the simulated data as well as the theoretical PDF of the 10-cell mixture. The following code produces this figure: 
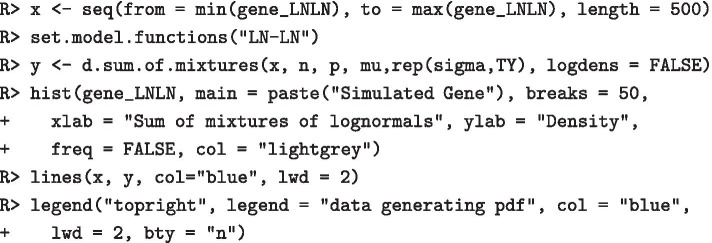

#### Parameter estimation

Next, we show how the parameters used above can be back-inferred from the generated dataset using maximum likelihood estimation. 
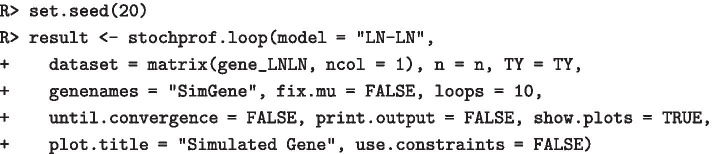


**Fig. 5:**
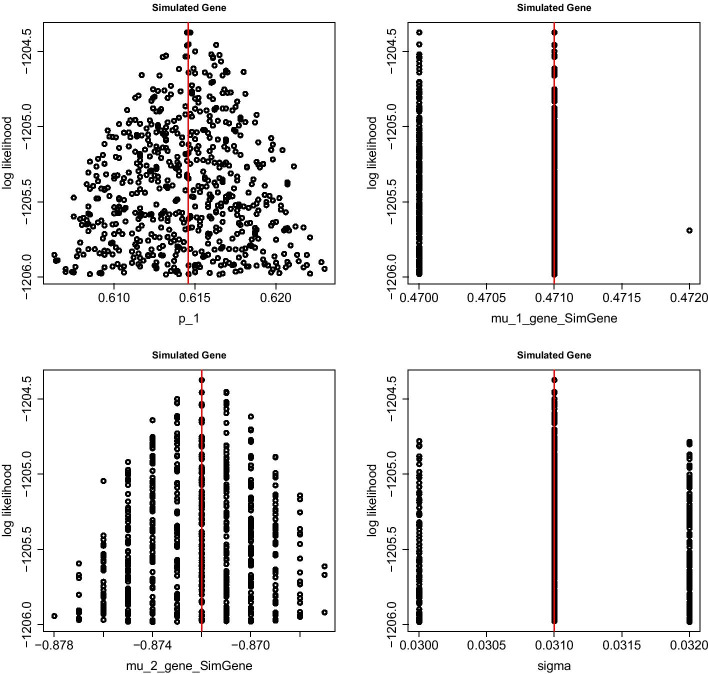
Graphical output of the parameter estimation procedure for $p,\,\mu _1,\,\mu _2\,\text{and}\,\sigma$ as described in Section Parameter estimation. Each point in the plots corresponds to one combination of values for $p,\,\mu _1,\,\mu _2\,\text{and}\,\,\sigma$. Each plot depicts the functional relationship between one parameter (e.g. $p$ in the upper left panel) and the log-likelihood function, whilst the remaining three parameters are integrated out

When the fitting is done, pressing <enter> causes R to show plots of the estimation process, see Fig. 5, and displays the results in the following form. 
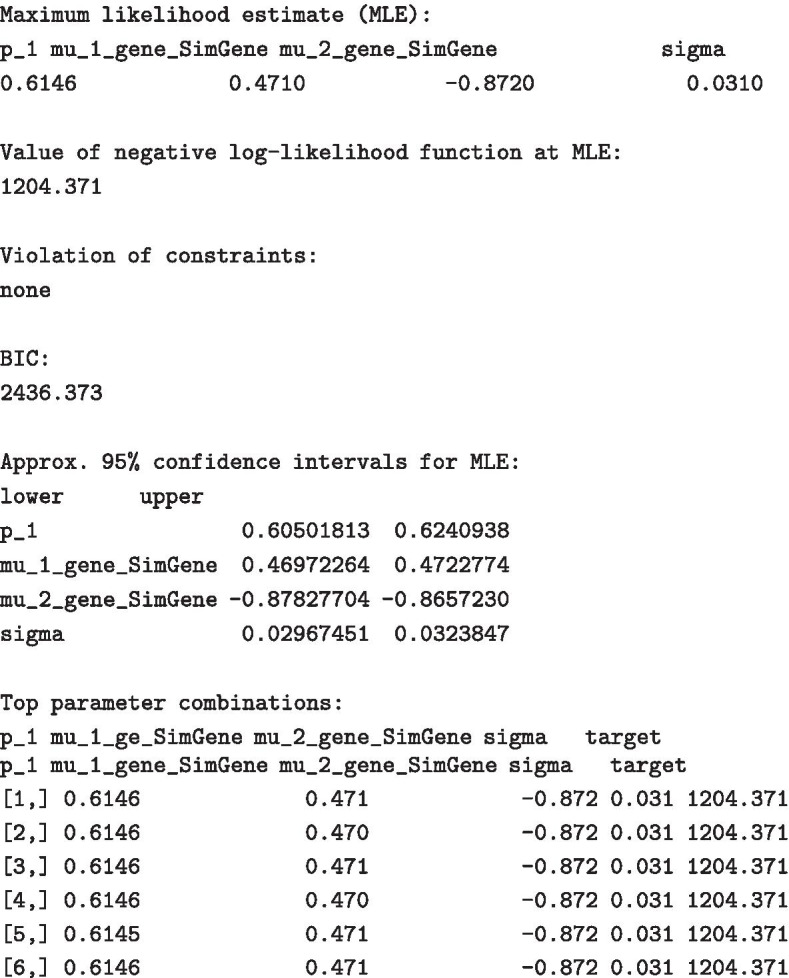
 Hence, the marginal confidence intervals cover the true parameter values.

### Simulation study on optimal pool size

Stochastic profiling, i.e. the analysis of small-pool gene expression measurements, is a compromise between the analysis of single cells and the consideration of large bulks: Single-cell information is most immediate, but a fixed number *k* of samples will only cover *k* cells. In pools of cells, on the other hand, information is convoluted, but *k* pools of size *n* cover *n* times as much material. An obvious question is the optimal pool size *n*. The answer is not available in analytically closed form. We hence study this question empirically.

For this simulation study, first, we generate synthetic data for different pool sizes with identical parameter values and settings. Then, we re-infer the model parameters using the stochprofML algorithm. This is repeated 1000 times for each choice of pool size, enabling us to study the algorithm’s performance by simple summary statistics of the replicates.

**Fig. 6:**
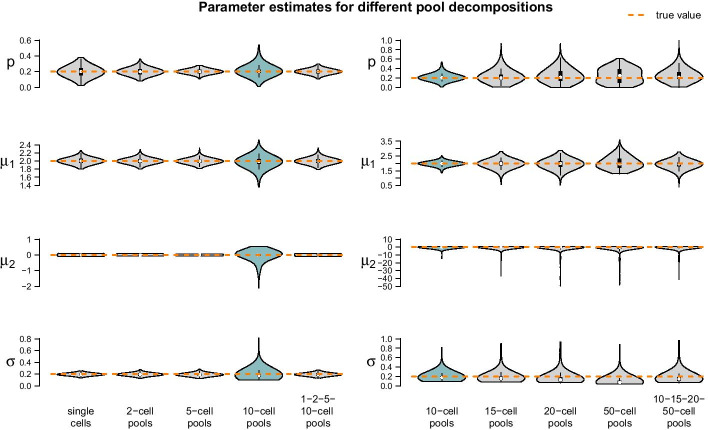
Violin plots of parameter estimates for two-population LN–LN model on 9000 simulated datasets, i.e. on 1000 datasets for each pool size composition. Left: Results for single-cell, 2-cell, 5-cell, 10-cell pool and their mixture. Right: Results for larger pool sizes, namely 10-, 15-, 20-, 50-cell pools and their mixture. Turquoise: Results for 10-cell pools; these are repeated across the left and right panels. The true parameters are marked in orange

The fixed settings are as follows: We use the two-population LN–LN model to generate data for one gene with $p_1 = 0.2$, $\mu _1 = 2$, $\mu _2 = 0$ and $\sigma = 0.2$. For each pool size we simulate $k=50$ observations. The pool sizes are chosen in nine different ways: In seven cases, pool sizes are identical for each sample, namely $n\in \{1,2,5,10,15,20,50\}$. In two additional cases, pool sizes are mixed, i.e. each of the *k* samples within one dataset represents a pool of different size $n_i\in \{1,2,5,10\}$ or $n_i\in \{10,15,20,50\}$. Figure 6 summarizes the point estimates of the 1000 datasets for each of the nine pool size settings. It seems that (for this particular choice of model parameter values) parameter estimation works reliably for pool sizes up to ten cells, with smaller variance from single-cells to 5-cells. This applies also for the mixture of pool sizes for the small cell numbers. For cell numbers larger than ten, the range of estimated values becomes considerably larger, but without obvious bias, which also applies to the mixture of the larger pool sizes. Additional file 6 shows repetitions of this study for different choices of population parameters. The results there confirm the observations made here.

Figure 6 suggests $n=5$ or varying small pool sizes as ideal choices since its estimates show smaller variance than the other pool sizes. This simulation study, however, has been performed in an idealized *in silico* setting: We did not include any measurement noise. In practice, however, it is well known that single-cells suffer more from such noise than samples with many cells. The ideal choice of pool size may hence be larger in practice.

### Simulation study on impact of parameter values

The underlying data-generating model obviously influences the ability of the maximum likelihood estimator to re-infer the true parameter values: Values of $p_1$ close to 0.5, small differences between $\mu _1$ and $\mu _2$ and large $\sigma$ blur the data and complicate parameter inference in practice. In the simulation study of this section, we investigate the sensitivity of parameter inference and which scenarios could be realistically identified.

We use the same datasets as in the previous simulation study: The parameter choices from before are considered as the standard and compared to those from Additional file 6. In detail, $p_1$ is reduced from 0.2 to 0.1 in one setting and increased to 0.4 in the next. $\mu _2$ is increased from 0 to 1, and $\sigma$ increases from 0.2 to 0.5. $\mu _1$ is kept fixed to 2 in all settings. As before, we consider 1000 data sets for every parameter setting and compare the resulting estimates to the true values. This was done for all pool sizes considered before, but here we only comment on the results of the 10-cell pools and refer to Additional file 6 for all other pool size settings.

**Fig. 7:**
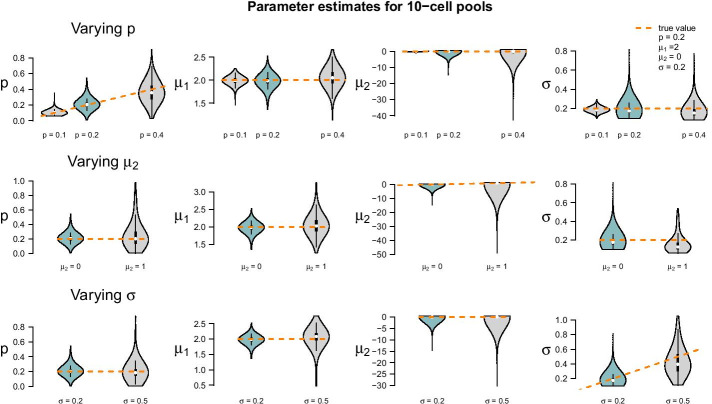
Violin plots of parameter estimates for two-population LN–LN model for varying parameters $p,\,\mu _2\,\text{and}\,\,\sigma$. Five parameter sets (see Additional file 6) were used to simulate 1000 datasets from each of which they were back-inferred. Violin plots for the standard setting $p=0.2,\,\mu _1 = 2,\,\mu _2 = 0\,\text{and}\,\sigma = 0.2$ are coloured turquoise. The true parameters used to simulate the data are marked in orange

Figure 7 shows the results of the study. In each row of the plot, we compare the estimates of the datasets that were simulated with the standard parameters to the estimates of the datasets that were simulated with one of the parameters changed. Even if only one parameter is changed all parameters are estimated. Each violin accumulates the estimates of 1000 datasets. For easier comparison, each of the twelve tiles shows the standard setting as turquoise violin, which means those are repeated in each row.

When changing the parameter values, they can still be derived without obvious additional bias, but accuracy decreases for increasing *p*, decreasing $\mu _2-\mu _1$ and increasing $\sigma$ (with few exceptions). Results for other pool sizes (see Additional file 6) show that these observations can be confirmed with some additions: Larger pool sizes infer parameters more accurately if *p* is smaller. In an increased first population setting ($p = 40\%$), $\mu _1$ can be better inferred if the data set consists of smaller pools. For larger pools, the estimation of $\mu _1$ and $\mu _2$ works comparably well after increasing $\mu _2$. In general, the estimation of $\sigma$ is the most difficult one: As shown in Eq. (1), the mean (and variance) of the lognormal distribution is determined by both the parameters $\mu _1$ and $\mu _2$ and by $\sigma$. Estimates of $\sigma$ will be negatively correlated with estimates ${\hat{\mu }}_1$ and ${\hat{\mu }}_2$ if the mean is determined correctly. Indeed, in pools of 15 cells with increased $\sigma$, we see that $\mu _1$ is slightly overestimated. Therefore, to keep the mean $\sigma$ is underestimated. This worsens in larger pools.

### Simulation study on the uncertainty of pool sizes

One key assumption of the stochprofML algorithm is that the exact number of cells in each cell pool is known. In [8], accordingly, ten cells were randomly taken from each sample by experimental design. However, different experimental protocols may not reveal the exact cell number: In [19], for example, tissue samples were taken as whole cancer spheroids. Here, the cell numbers were experimentally unknown but estimated using light sheet microscopy and 3D image analysis. Since the stochprofML algorithm requires the pool sizes as input parameter, some estimate has to be passed to it. It is intuitively obvious that the better the prior knowledge about the cell pool sizes, the better the final model parameter estimate. In this simulation study, we investigate the consequences of misspecification.

**Fig. 8:**
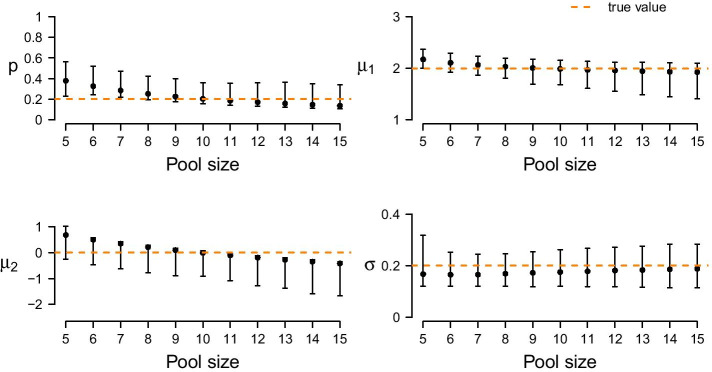
Parameter estimates for (partly) misspecified pool sizes across 1000 synthetic datasets: The true pool size is 10 in every dataset. The stochprofML algorithm, however, uses values from 5 to 15 as input parameter. Bars cover the range between the empirical 2.5%- and 97.5%-quantiles. The dots mark the empirical median, the orange line the true parameter values used for simulation

**Fig. 9:**
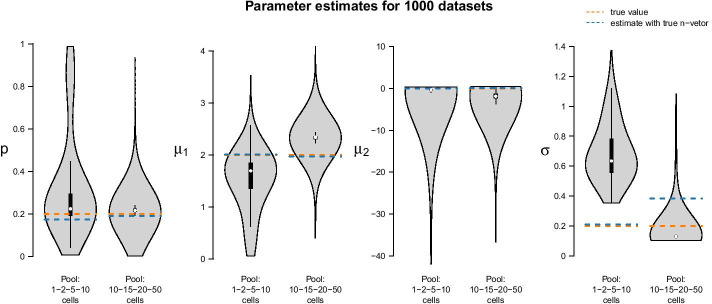
Parameter inference under misspecification of the cell pool size: parameters are estimated for two datasets, one generated based on a pool size vector $\vec{n}_\text{small}{}$ with values between 1 and 10 (left violin in each panel); the other one based on a vector $\vec{n}_\text{large}{}$ with values between 10 and 50 (right violin in each panel). From left to right: Estimates of $p,\,\mu _1,\,\mu _2\,\text{and}\,\,\sigma$. The violins depict estimates across 1000 estimation runs, where each relies on a randomly sampled misspecified pool size vector as described in the main text. Orange: True parameters values. Light blue: Estimates without misspecification of the pool size vector

In a first simulation study, we reuse from before the 1000 synthetic 10-cell datasets. Each of these contains 50 10-cell samples, simulated with underlying model parameters $p = 0.2$, $\mu _1 = 2$, $\mu _2 = 0$ and $\sigma = 0.2$. As before, we re-infer the population parameters using the stochprofML algorithm. This time, however, we use varying pool sizes from 5 to 15 as input parameters of the algorithm. This is a misspecification except for the true value 10. The resulting parameter estimates (empirical median and 2.5%-/97.5%-quantiles across the 1000 datasets) are depicted in Fig. 8. Estimates are optimal or at least among the best in terms of empirical bias and variance when using the correct pool size. With increasing assumed cell number, the estimates of *p* decrease, i.e. the fraction of cells from the higher expressed population is assumed to be smaller. This is a reasonable consequence of overestimating *n*, because in this case the surplus cells are assigned to the second population with lower (or even close-to-zero) expression. Consequently, at the same time the estimates of $\mu _2$ decrease to be even smaller. In a second simulation study, we use the two settings with mixed cell pool sizes as introduced above. One setting embraces cell pools with rather small cell numbers (single-, 2-, 5- and 10-cell samples), the other one pools with larger cell numbers (10-, 15-, 20- and 50-cell samples). For each of the two scenarios, we generate one dataset with 50 samples. We denote the true 50-dimensional pool size vectors by $\vec{ n}_\text{small}$ and $\vec{ n}_\text{large}$ and employ these vectors for re-estimating the model parameters *p*, $\mu _1$, $\mu _2$ and $\sigma$. Then, we estimate the parameters again for the same two datasets for 1000 times, but this time using perturbed pool size vectors as input to the algorithm, introducing artificial misspecification. These 50-dimensional pool size vectors are generated as follows: For each component, we draw a Poisson-distributed random variable with intensity parameter equal to the respective component of the true vectors $\vec{ n}_\text{small}$ or $\vec{ n}_\text{large}$. Zeros are set to one, the minimum pool size. Figure 9 shows these $2\times 1000$ parameter estimates as compared to the true parameter values and those for which the true size vectors $\vec{ n}_\text{small}$ and $\vec{ n}_\text{large}$ were used as input. The violins of the estimates for the smaller cell pools (based on $\vec{n}_\text{small}$) indicate that the estimates of *p* and $\mu _1$ are fairly accurate, but the estimates of $\mu _2$ have large variance, and $\sigma$ is overestimated in all 1000 runs. This is plausible as population 1 (the one with higher log-mean gene expression) is only present on average in 20% of the cells; even when misspecifying the pool sizes, the cells of population 1 are still detectable since this is the population responsible for most gene expression. Consequently, all remaining cells are assigned to population 2, which has lower or even almost no expression. If the pool size is assumed too low, this second population will be estimated to have on average a higher expression; if it is assumed too large, the second population will be estimated to have a lower expression. This leads to a broader distribution and thus an overestimation of $\sigma$.

The results for the larger cell pools (based on $\vec{n}_\text{large}$) show a similar pattern. In this case, however, the impact of misspecification is less visible, as also confirmed by additional simulations in Additional file 6. For large cell pools, the averaging effect across cells is strong anyway and in that sense more robust. In the study here, due to variability of parameter estimates, the $\sigma$ parameter is often even better estimated when using a misspecified pool size vector than when using the true one. It might also be appropriate to repeat the parameter estimation, as shown here, with similar pool size vectors to get more robust estimates.

Taken together, stochprofML can be used even if exact pool sizes are unknown. In that case, the numbers should be approximated as well as possible.

### Interpretation of estimated heterogeneity

The stochprofML algorithm estimates the assumed parameterized single-cell distributions underlying the samples and, as described before, selects the most appropriate number of cell populations using the BIC. Assume we have performed this estimation for samples from two different groups, cases and controls. One may in practice then want to know whether the inferred single-cell populations are substantially different between the two groups, e.g. in case the estimated log-means ${\hat{\mu }}_\text{cases}$ and ${\hat{\mu }}_\text{controls}$ are close to each other. A related question is whether the difference is biologically relevant.

We hence seek a method that can judge statistical significance and potentially reject the null hypothesis that two single-cell populations are the same; and at the same time allow the interpretation of similarity. Direct application of Kolmogorov-Smirnov or likelihood-ratio tests to the observed data is impossible here since the single-cell data is unobserved: We only measure the overall gene expression of pools of cells. Calculation of the Kullback-Leibler divergence of the two distributions would be possible; however, it is not target-oriented for our application where we seek an interpretable measure of similarity rather than a comparison between more than two population densities.

**Fig. 10:**
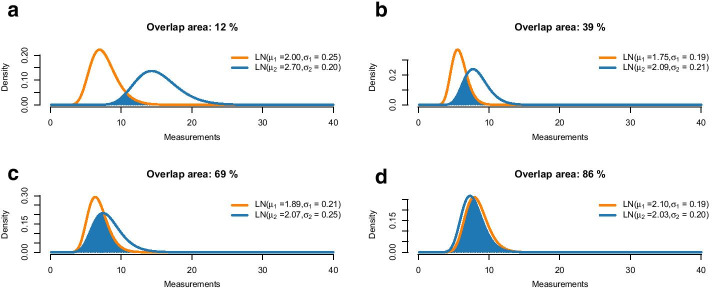
Four examples of overlapping PDFs, together with the overlap area as defined in Eq. (7).  Slightly different parameters result in very small overlap area such as 12% in **a** or 39% in **b**. An overlap area of 69% in **c** still shows two very different distributions. Overlaps larger than 86% as in **d** come with similar distributions

For our purposes, we use a simple intuitive measure of similarity—the overlap of two PDFs, that is the intersection of the areas under both PDF curves:

7 $$\begin{aligned} \text{OVL}(f,g)=\int _{-\infty }^\infty \min \{f(x),g(x)\}dx \end{aligned}$$

for two continuous one-dimensional PDFs *f* and *g* [see also 25]. The overlap lies between zero and one, with zero indicating maximum dissimilarity and one implying (almost sure) equality. In our case, we are particularly interested in the overlap of two lognormal PDFs: 
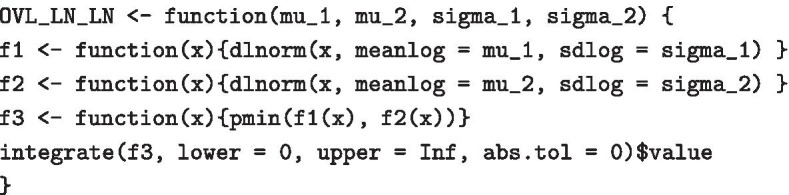


Figure 10 shows examples of such overlaps. Here, the overlap ranges from 12% for two quite different distributions to 86% for two seemingly similar distributions. The question is where to draw a cutoff, that is, at what point we decide to label two distributions as different. Current literature considers two cases: Either the parametric case [e.g. 26], where both distributions are given by their distribution families and parameter values; or the nonparametric case [e.g. 25], where observations (but no theoretical distributions) are available for the two populations. Our application builds a third case: On the one hand, we want to compare two parametric distributions, but the model parameters are just given as estimates based on (potentially small) datasets, thus they are uncertain; on the other hand, we do not directly observe the single-cell gene expression but just the pooled one. To address this issue, we suggest to again take into account the original data that led to the estimated parametric PDFs. As an example, assume that we consider two sets of pooled gene expression, one for a group of cases and one for a group of controls. In both groups, pooled gene expression is available as 10-cell measurements, but the two groups differ in sample size. Let’s say the cases contain 50 samples and the controls 100. We assume the LN–LN model with two populations and estimate the mixture and population parameters using the stochprofML algorithm separately for each group, leading to estimates $\hat{p}_\text{cases},\,\hat{\mu }_{1,\text{cases}},\,\hat{\mu }_{2,\text{cases}},\,\hat{\sigma }_\text{cases}{} \,\text{and}\,\hat{p}_\text{controls},\,\hat{\mu }_{1,\text{controls}},\,\hat{\mu }_{2,\text{controls}},\,\hat{\sigma }_\text{controls}{}$. We now aim to assess whether the first populations in both groups have identical characteristics, i.e. whether ${\mathcal{L}}{\mathcal{N}}(\hat{\mu }_{1,\text{cases}},\hat{\sigma }^2_\text{cases})\,\text{and}\,\,{\mathcal{L}}{\mathcal{N}}(\hat{\mu }_{1,\text{controls}},\hat{\sigma }^2_\text{controls})$ are estimates of the same distribution.

Figure 10 displays the single-cell PDFs of the first population and their overlaps for various values of the estimates. For example, in Fig. 10d, the orange curve shows the single-cell PDF of population 1 inferred from the cases, yielding ${\mathcal{L}}{\mathcal{N}}(\hat{\mu }_{1,\text{cases}}=2.10,\hat{\sigma }^2_\text{cases}=0.19^2)$, and the blue one shows the inferred single-cell PDF of population 1 from the controls, ${\mathcal{L}}{\mathcal{N}}(\hat{\mu }_{1,\text{controls}}=2.03,\hat{\sigma }^2_\text{controls}=0.20^2)$. The overlap of these two inferred PDFs equals 86%.

We now aim to test the null hypothesis that the underlying populations

${\mathcal{L}}{\mathcal{N}}({\mu }_{1,\text{cases}},{\sigma }^2_\text{cases})\,\text{and}\,\,{\mathcal{L}}{\mathcal{N}}({\mu }_{1,\text{controls}},{\sigma }^2_\text{controls})$ are the same versus the experimental hypothesis that they are different. We perform a sampling-based test: Taking into account the inferred population probabilities $\hat{p}_\text{cases}{} \,\text{and}\,\hat{p}_\text{controls}$ and the number of samples and cells in the data, we can estimate the number of cells which the estimates $\hat{\varvec{\theta }}_\text{cases}\, \text{and}\,\hat{\varvec{\theta }}_\text{controls}$ relied on. The larger this cell number, the less expected uncertainty about the estimated population distributions ${\mathcal{L}}{\mathcal{N}}(\hat{\mu }_{1,\text{cases}},\hat{\sigma }^2_\text{cases})\,\text{and}\,\,{\mathcal{L}}{\mathcal{N}}(\hat{\mu }_{1,\text{controls}},\hat{\sigma }^2_\text{controls})$ (neglecting the impact of pool sizes). In our example, let $\hat{p}_\text{cases}=12\%$. Then, approximately 12% of the 500 cells from the cases group ($50 \times$ 10-cell samples) belonged to population 1, that is 60 cells. For $\hat{p}_\text{controls}=20\%$, 200 cells were expected to be from the first population (that is 20% of 1000 cells, coming from the 100 $\times$ 10-cell measurements for the controls). In our procedure, we compare parameter estimates that are based on the respective numbers of single cells, i.e. 60 cells for cases and 200 cells for controls. We perform the following steps:

- Calculate $\text{OVL}_{\text{original}},$ the overlap of the PDFs of ${\mathcal{N}}(\hat{\mu }_{1,\text{cases}}=2.10,\hat{\sigma }^2_\text{cases}=0.19^2)\,\text{and}\,{\mathcal{L}}{\mathcal{N}}(\hat{\mu }_{1,\text{controls}}=2.03,\hat{\sigma }_\text{controls}^2=0.20^2)$.
- Under the null hypothesis, the two distributions are identical. We approximate the parameters of this identical distribution as $\tilde{\mu }_{1,\text{mean}}= (\hat{\mu }_{1,\text{cases}}+\hat{\mu }_{1,\text{controls}})/2\,\text{and}\,\tilde{\sigma }_\text{mean}= (\hat{\sigma }_\text{cases}+\hat{\sigma }_\text{controls})/2$.
- Repeat $$N = 1000$$ times:
  - Draw dataset *A* of size 60 from ${\mathcal{L}}{\mathcal{N}}(\tilde{\mu }_{1,\text{mean}},\tilde{\sigma }_\text{mean}^2)$.
  - Draw dataset *B* of size 200 from ${\mathcal{L}}{\mathcal{N}}(\tilde{\mu }_{1,\text{mean}},\tilde{\sigma }_\text{mean}^2)$.
  - Estimate the log-mean and log-sd for these two datasets using the method of maximum likelihood, yielding $\hat{\mu }_A,\,\hat{\sigma }_A,\,\hat{\mu }_B\,\text{and}\,\hat{\sigma }_B$.
  - Calculate $$\text{OVL}\left( f_{{\mathcal{L}}{\mathcal{N}}(\hat{\mu }_A,\hat{\sigma }_A^2)},f_{{\mathcal{L}}{\mathcal{N}}(\hat{\mu }_B,\hat{\sigma }_B^2)}\right)$$.
- Sort the $N$ overlap values and select the empirical 5% quantile $\text{OVL}_{0.05}{}$.
- Compare the overlap from the original data to this quantile:
  - If $\text{OVL}_{original}\le \text{OVL}_{0.05}{}$, the null hypothesis that both populations are the same can be rejected.
  - If $\text{OVL}_{original}>\text{OVL}_{0.05}{}$, the null hypothesis cannot be rejected.

This procedure is related to the idea of parametric bootstrap and the bootstrap percentile method with the difference that our original data is on the $n$-cell level and the parametrically simulated data is on the single-cell level. Note that under the null hypothesis the two population distributions are identical, but in practice this will hardly ever be the case for the estimated population parameters. Taking the average of estimated log-means and log-standard deviations in the second bullet point above is one way of approximating the null distribution.

**Fig. 11:**
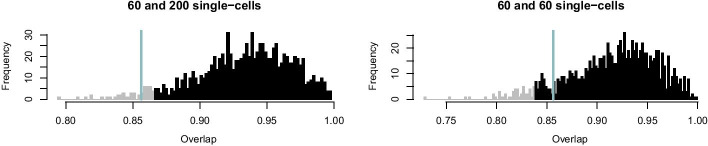
Variability of the overlap between the PDFs of the two distributions described in Fig. 10d. The panels show histograms of $N=1000$ simulated overlap values which are simulated as described in the main text. Left: We assume that the estimates of the orange distribution relied on 60 single cells and the blue distribution on 200 single cells. Right: For both distributions, parameters are assumed to be estimated on 60 single cells. The 86% overlap of the original PDFs from Fig. 10d, i.e. ${\mathcal{L}}{\mathcal{N}}({\hat{\mu }}_{1,cases}=2.10,{\hat{\sigma }}^2_{cases}=0.19^2)\,\text{and}\,{\mathcal{L}}{\mathcal{N}}({\hat{\mu }}_{1,controls}=2.03,{\hat{\sigma }}_{controls}^2=0.20^2)$, is marked in turquoise. The light grey bars of the histogram indicate values below the empirical 5%-quantile. If the original overlap falls into this range, we reject the null hypothesis that both distributions identical

The left panel of Fig. 11 shows one outcome of the above-described procedure (i.e. the stochastic, sampling-based algorithm was run once) with the above-specified values of the parameter estimates. Here, $\text{OVL}_{original}{}$ lies in the critical range such that we reject the null hypothesis that the gene expression of the populations in question stem from the same lognormal distribution. We thus assume a difference here. The right panel of Fig. 11 demonstrates the importance of taking into account the number of cells which the original estimates were based on: Here, we show one outcome of the above described steps, but this time we assume that for the control group there were only 30 10-cell samples (i.e. 300 cells in total). With the same population fraction as before ($\hat{p}_\text{controls}=20\%),$ the datasets *B* now contain only 60 cells. Here, the value $\text{OVL}_{original}{}$ does not fall into the critical range, and therefore we would not reject the null hypothesis that the two populations of interest are the same.

When testing for heterogeneity for several genes simultaneously, multiple testing issues should be taken into account. However, genes will not in general be independent from each other.

### Prediction of sample compositions

**Fig. 12:**
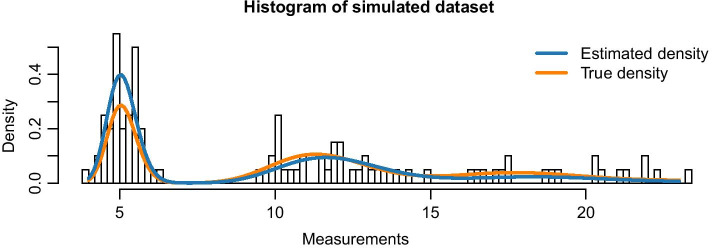
Histogram of simulated data underlying the prediction of cell pool compositions in Fig. 13a: 100 synthetic 5-cell measurements arising from the LN–LN model with two populations with parameters $\varvec{p}=(0.2,0.8),\,\varvec{\mu }=(2,0)\,\text{and}\,\,\sigma =0.2$. The PDF with true model parameters is shown in orange, the PDF with estimated parameters $\varvec{\hat{p}}=(0.14, 0.86),\,\varvec{\hat{\mu }}=( 2.04, 0)\,\text{and}\,\hat{\sigma }= 0.20$ in blue

The stochprofML algorithm estimates the parameters of the mixture model, i.e.—in case of at least two populations—the probability for each cell within a pool to fall into the specific populations. It does *not* reveal the individual pool compositions. In some applications, however, exactly this information is of particular interest. Here, we present how one can infer likely population compositions of a particular cell pool. This is done in a two-step approach via conditional prediction: First, one estimates the model parameters from the observed pooled gene expression, i.e. one obtains an estimate $\hat{\varvec{\theta }} \,\text{of}\,{\varvec{\theta }}.$ Then, one assumes that $\varvec{\theta }{} \text{ equals}\,\hat{\varvec{\theta }}{}$ and derives the most probable population composition via maximizing the conditional probability of a specific composition given the pooled gene expression (for calculations, see Additional file 4). We evaluate this procedure via a simulation study. As before, we simulate data using the stochprofML package. In particular, we use the LN–LN model with two populations with parameters $\varvec{p}=(0.2,0.8),\,\varvec{\mu }=(2,0)\,\text{and}\,\sigma =0.2$ . Each simulated measurement shall contain the pooled expression of *n* = 5 cells, and we sample k = 100 such measurements. We store the original true cell pool compositions from the data simulation step in order to later compare the composition predictions to the ground truth. Having generated the synthetic data, we apply stochprofML to estimate the model parameters $\varvec{p},\,\varvec{\mu } \,\text{and}\,\sigma$. Figure 12 shows a histogram of one simulated data set along with the PDF of the true population mixture and the PDF of the estimated population mixture (that is the LN–LN model with parameters $\varvec{\hat{p}}=(0.14, 0.86),\,\varvec{\hat{\mu }}=( 2.04, 0)\,\text{and}\,\hat{\sigma }= 0.20$).

**Fig. 13:**
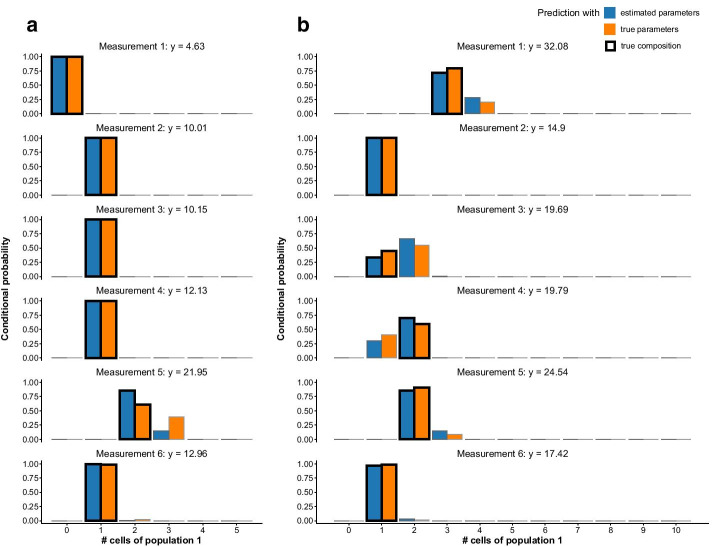
Estimation of cell pool compositions in the two-population LN–LN model: conditional probabilities of numbers of cells from the first population in the first six measurements of the synthetic datasets described in the main text and in Fig. 12, given the respective pooled gene expression measurement. Blue bars show the conditional probabilities using estimated model parameters, and orange bars show those when using the true parameters. True cell numbers from the first population are marked with a black box around the bars. Results for **a** simulated 5-cell data and, **b** 10-cell data

Next, we calculate the conditional probability mass function (PMF; see Additional file 4 for details) for each possible population composition conditioned on the particular pooled gene expression measurement. Figure 13a and Table 2 show results for the first six (out of 100) pooled measurements.

In particular, Fig. 13a displays the conditional PMF of all possible compositions (i.e. *k* times population and 5-*k* times population 2 for $k\in \{0,1,\ldots ,5\}).$ Blue bars stand for these probabilities when $\hat{\varvec{\theta }}$ is used as model parameter value. Orange stands for the hypothetical case where the true value $\varvec{\theta }{}$ is known and used. These two scenarios are in good agreement with each other.

**Table 2:** Estimates of numbers of cells from the first population in the simulated 5-cell data described in Figs. 12 and 13a and in the main text

Estimator for # of cells in pop. 1		Measurement index						# of hits
		1	2	3	4	5	6	
Estimated parameters	Mean	0.00	1.00	1.00	1.00	2.14	1.01	98
	MLE (CI)	0 (0,0)	1 (1,1)	1 (1,1)	1 (1,1)	2 (2,3)	1 (1,1)	98 (100)
True parameters	Mean	0.00	1.00	1.00	1.00	2.39	1.02	97
	MLE (CI)	0 (0,0)	1 (1,1)	1 (1,1)	1 (1,1)	2 (2,3)	1 (1,1)	97 (100)
True # of cells from population 1		0	1	1	1	2	1	

*Columns:* Estimation results for the first six measurements from the datasets and (last column) summary across all 100 samples. *Rows:* Estimation of cell numbers are based on conditional probabilities that use either the estimated model parameters (rows 1 and 2, corresponding to blue bars in Fig. 13a) or the true values (rows 3 and 4, orange bars). Within each of these two choices one can consider the mean number of cells from population 1 as determined by the conditional probabilities (rows 1 and 3) or the maximum likelihood estimator (MLE) that maximizes the conditional probabilities (rows 2 and 4, first value) including a 95% confidence interval that covers at least 95% of the conditional probability mass (rows 2 and 4, in parentheses). The last row shows the true pool composition. The last column shows for each estimator how many of the 100 cell numbers were inferred correctly (defined as follows: rounded mean is exact match; MLE is exact match; confidence interval (CI) includes correct number)

We regard the most likely sample composition to be the one that maximizes the conditional PMF (maximum likelihood principle). The true composition (ground truth) is marked with a black box around the blue and orange bars. We observe in Fig. 13a that the composition is in all six cases inferred correctly and mostly unambiguously. Only for the fifth measurement, there is visible probability mass on a composition other than the true one. In fact, it is the only pool (out of the six considered ones) with two cells from the first population. Alternatively to the maximum likelihood estimator, one can also regard the expected composition—the empirical weighted mean of numbers of cells in the first population—or confidence intervals for this number. The respective estimates for the first six measurements of the dataset are shown in Table 2. The results are consistent with the interpretation of Fig. 13a. Certainly, the precision of the prediction depends on the employed pool sizes, the underlying true model parameters and how reliably these were inferred during the first step. We showed above that larger cell pools lead to less precise parameter inference. Hence, we repeat the prediction of sample compositions on another dataset, this time based on $10$-cell pools. All other parameters remain unchanged. The resulting conditional probabilities are depicted in Fig. 13b. Since $p=0.2$, one expects on average two cells to be from the first population in each 10-cell pool. As in the previous $5$-cell case, most predictions show a clear pattern. However, probability masses are spread more widely. Measurements 3 and 4 exemplify that almost identical gene expression measurements ($y=19.69\,\text{and}\,y=19.79$) can arise from different underlying pool compositions (two times population 1 in measurement 3 vs. three times population 1 in measurement 4). For more similar population parameters, the estimation will get worse, which will then propagate to the well composition prediction. In such cases, to predict the pool compositions, one may use additional parallel measurements of other genes that might separate the population better by their different expression profiles while the pool composition stays the same across genes.

## Conclusion

With the stochprofML package, we provide an environment to profile gene expression measurements obtained from small pools of cells. Experimentalists may choose this approach if single-cell measurements are impossible in their lab (e.g. for bacteria), if the drop-out rate is high in single-cell libraries, if budget or time are limited, or if one prefers to avoid the stress which is put on the cells during separation. The latest implementation even allows to combine information from different pool sizes, in particular, to simultaneously analyze single-cell and and $n$-cell data.

We demonstrated the usage and performance of the stochprofML algorithm in various examples and simulation studies. These have been performed in an idealized *in silico* environment. This should be kept in mind when incorporating the results into experimental planning and analysis. Subsequent interpretation of heterogeneity will be informative if based on a good model estimate. The assumption of independent expression across genes within the same tissue sample is a simplification of nature that leads to less complex parameter estimation. Previous experimental validation [9] provided evidence that transcriptional heterogeneity can be parameterized through stochastic profiling even for non-ideal settings such as small sample sizes or in the presence of gene-gene correlation. If populations are similar or diffuse, they may not be identified as distinct populations through stochprofML. The same, however, applies to other statistical methods and also to the analysis of single-cell data. For the latter, noise is expected to be more pronounced than in $n$-cell pools, which again motivates the use of our method.

The optimal pool size with respect to bias and variance of the corresponding parameter estimators will depend on unknown properties such as numbers of populations and their characteristics, and also on the relationship between the pool size and the amount of technical measurement noise. The latter aspect has been excluded from the studies here but further supports the application of stochastic profiling.

## Availability of data and requirements

**Project name:** stochprofML.

**Project home page:** https://github.com/fuchslab/stochprofML.

**Operating system(s):** Platform independent.

**License:** GNU GPL.

**Programming language:** R.

**Other requirements:** None; We used R version 3.5.3 [27]. In addition to our stochprofML version 2.0.3 [28], we attached the following R packages: MASS version 7.3-51.1 [29], numDeriv version 2016.8-1.1 [30], EnvStats version 2.3.1 [31], vioplot version 0.3.4. [32], zoo version 1.8-7 [33], sm version 2.2-5.6 [34], cowplot version 1.0.0 [35], ggplot2 version 3.2.1 [36], knitr version 1.27 [37], microbenchmark version 1.4-7 [38], and RcolorBrewer version 1.1-2 [39]. All calculations were performed on a 64-bit x86_64-redhat-linux-gnu platform running under Fedora 28.

**Any restrictions to use by non-academics**: GPL license, open source.

## Supplementary information

**Additional file 1: PDF of n-cell measurements of T cell populations**. Derivation of the PDF shown in Eq. (3) in Section Small-pool models of heterogeneous gene expression.

**Additional file 2: PDF of pooled gene expression for mixed pool size vectors**. Derivation of the PDF of samples that contain different cell numbers.

**Additional file 3: Transformation of population probabilities**. Details about the transformation of the population probabilities during parameter optimization.

**Additional file 4: Derivation of sample composition probabilities**. Derivation of the conditional probability of a cell composition given the measured gene expression needed in Section Prediction of sample compositions.

**Additional file 5: Interactive Functions**. Examples how the interactive usage of stochprofML works.

**Additional file 6: Details on Simulation Studies**. More information and further details on the Simulation study on optimal pool size, on the Simulation study on impact of parameter values and on the Simulation study on the uncertainty of pool sizes.

## Data Availability

All scripts used in this study can be found in our open GitHub repository https://github.com/fuchslab/Stochastic_Profiling_in_R.

## Abbreviations

mRNA: Messenger ribonucleic acid
LN–LN model: Lognormal–lognormal model
rLN–LN model: Relaxed lognormal–lognormal model
EXP–LN model: Exponential–lognormal model
PDF: Probability density function
BIC: Bayesian information criterion
OVL: Overlap
PMF: Probability mass function
MLE: Maximum likelihood estimator
CI: Confidence interval
