# Supplementary material for: stochprofML: stochastic profiling using maximum likelihood estimation in R
Source: BMC Bioinformatics. 2021 Mar 15;22:123. doi: 10.1186/s12859-021-03970-7 (PMC7958472; doi:10.1186/s12859-021-03970-7)
Supplement: Supplementary file 1 — Additional file 1: PDF of n-cell measurements of T cell populations. Derivation of the PDF shown in Eq. (3) in Section Small-pool models of heterogeneous gene expression. [file 12859_2021_3970_MOESM1_ESM.pdf]

stochprofML: stochastic profiling using maximum likelihood estimation in R

Lisa Amrhein and Christiane Fuchs

Additional File 1

PDF of n-cell measurements of T cell populations

Equation (3) in the Section Small-pool models of heterogeneous gene expression of the main text displays the PDF of the overall gene expression of a cell pool of size n_i, where each single cell from the pool can originate from any of T cell populations. We derive this PDF here. To make it easier to follow the lengthy calculations, we build up the formulas in four steps: We start with the simplest case of 2-cell measurements in the presence of two populations. Then, we continue with 2-cell samples and three populations. Next, we increase the cell number to n and finally raise the population number to T.

PDF of 2-cell measurements of two populations ($n = 2$, $T = 2$)

First, we derive the PDF of a measurement y of a 2-cell pool, i.e. of $Y = X_1 + X_2$. Assume we know that two cell populations are present in the tissue, and each of them is described by an individual distribution. In this section, we denote the univariate population distributions by $\mathcal{D}_h$, $h = 1, \ldots, T = 2$. In the main text, they are replaced by the distributions that were presented in Section Single-cell models of heterogeneous gene expression: $\mathcal{LN}(\mu, \sigma^2)$ or $\mathcal{EXP}(\lambda)$ with population-specific parameter values. For now, we consider for $j = 1, 2$

$$X_j \overset{iid}{\sim} \begin{cases} \mathcal{D}_1 & \text{with probability } p_1 \\ \mathcal{D}_2 & \text{with probability } 1 - p_1, \end{cases}$$

where $p_1 \in [0, 1]$. Hence, the PDF of each X_j is

$$f_X(x) = p_1 f_{\mathcal{D}_1}(x) + p_2 f_{\mathcal{D}_2}(x)$$

with $p_2 = 1 - p_1$. To determine the distribution of Y, we use the convolution of the single-cell PDFs, which are the same functions f_X for both X_1 and X_2:

$$\begin{aligned}
f_Y(y) &= \int_0^y f_X(x_1) f_X(y - x_1) dx_1 \\
&= \int_0^y \left(\Big[p_1 f_{\mathcal{D}_1}(x_1) + p_2 f_{\mathcal{D}_2}(x_1) \Big] \Big[p_1 f_{\mathcal{D}_1}(y - x_1) + p_2 f_{\mathcal{D}_2}(y - x_1) \Big] \right) dx_1 \\
&= \int_0^y \Bigg(p_1^2 f_{\mathcal{D}_1}(x_1) f_{\mathcal{D}_1}(y - x_1) + p_2^2 f_{\mathcal{D}_2}(x_1) f_{\mathcal{D}_2}(y - x_1) \\
&\qquad + p_1 p_2 f_{\mathcal{D}_1}(x_1) f_{\mathcal{D}_2}(y - x_1) + p_2 p_1 f_{\mathcal{D}_2}(x_1) f_{\mathcal{D}_1}(y - x_1) \Bigg) dx_1 \\
&= p_1^2 \int_0^y f_{\mathcal{D}_1}(x_1) f_{\mathcal{D}_1}(y - x_1) dx_1 + p_2^2 \int_0^y f_{\mathcal{D}_2}(x_1) f_{\mathcal{D}_2}(y - x_1) dx_1 \\
&\qquad + p_1 p_2 \int_0^y f_{\mathcal{D}_1}(x_1) f_{\mathcal{D}_2}(y - x_1) dx_1 + p_2 p_1 \int_0^y f_{\mathcal{D}_2}(x_1) f_{\mathcal{D}_1}(y - x_1) dx_1.
\end{aligned}$$

Each of these integrals $\int_0^y f_{\mathcal{D}_i}(x_1) f_{\mathcal{D}_j}(y - x_1) dx_1$ is the PDF of a random variable $Z_1 + Z_2$ evaluated at y, where $Z_1 \sim \mathcal{D}_i$ and $Z_2 \sim \mathcal{D}_j$ are independent. This holds for both $i \neq j$ and $i = j$. We denote this density by $f_{i,j}$. All together, we get

$$f_Y(y) = \sum_{i=1}^{2} \sum_{j=1}^{2} p_i p_j f_{i,j}(y).$$

An alternative formulation is

$$f_Y(y) = \sum_{\ell_1=0}^{2} \binom{2}{\ell_1} p_1^{\ell_1} p_2^{\ell_2} f_{(\ell_1,\ell_2)}(y), \tag{1}$$

where ℓ_1 and $\ell_2 = 2 - \ell_1$ show how often a cell of population 1 and 2 is present in the pool. The two PDFs $f_{(\ell_1,\ell_2)}$ and $f_{i,j}$ are directly connected: $f_{(\ell_1,\ell_2)}$ considers *how often* populations 1 and 2 are represented, and $f_{i,j}$ denotes *which* populations are present. For example, $f_{(1,1)}(y) = f_{1,2}(y)$ and $f_{(0,2)}(y) = f_{2,2}(y)$.

PDF of 2-cell measurements of three populations ($n = 2$, $T = 3$)

Next, we derive the PDF of a measurement y of a 2-cell pool, i.e. of $Y = X_1 + X_2$. Now, we assume three cell populations to be present in the tissue. Again, each of them is described by an individual distribution $\mathcal{D}_h$ for $h = 1, \ldots, T = 3$:

$$X_j \overset{iid}{\sim} \begin{cases} \mathcal{D}_1 & \text{w.p. } p_1 \\ \mathcal{D}_2 & \text{w.p. } p_2 \\ \mathcal{D}_3 & \text{w.p. } 1 - p_1 - p_2, \end{cases}$$

for $j = 1, 2$ where $p_1, p_2 \in [0, 1]$ and $p_1 + p_2 \leq 1$. Hence, the PDF of each X_j is

$$f_X(x) = p_1 f_{\mathcal{D}_1}(x) + p_2 f_{\mathcal{D}_2}(x) + p_3 f_{\mathcal{D}_3}(x)$$

with $p_3 = 1 - p_1 - p_2$. To determine the distribution of $Y = X_1 + X_2$, we again use the convolution of the single-cell PDFs:

$$\begin{aligned} f_Y(y) &= \int_0^y f_X(x_1) f_X(y - x_1) dx_1 \\ &= \int_0^y \Bigg(\Big[p_1 f_{\mathcal{D}_1}(x_1) + p_2 f_{\mathcal{D}_2}(x_1) + p_3 f_{\mathcal{D}_3}(x_1) \Big] \\ &\qquad \times \Big[p_1 f_{\mathcal{D}_1}(y - x_1) + p_2 f_{\mathcal{D}_2}(y - x_1) + p_3 f_{\mathcal{D}_3}(y - x_1) \Big] \Bigg) dx_1 \\ &= \int_0^y \Bigg(p_1^2 f_{\mathcal{D}_1}(x_1) f_{\mathcal{D}_1}(y - x_1) \\ &\qquad + p_2^2 f_{\mathcal{D}_2}(x_1) f_{\mathcal{D}_2}(y - x_1) + p_3^2 f_{\mathcal{D}_3}(x_1) f_{\mathcal{D}_3}(y - x_1) \\ &\qquad + p_1 p_2 f_{\mathcal{D}_1}(x_1) f_{\mathcal{D}_2}(y - x_1) + p_2 p_1 f_{\mathcal{D}_2}(x_1) f_{\mathcal{D}_1}(y - x_1) \\ &\qquad + p_1 p_3 f_{\mathcal{D}_1}(x_1) f_{\mathcal{D}_3}(y - x_1) + p_3 p_1 f_{\mathcal{D}_3}(x_1) f_{\mathcal{D}_1}(y - x_1) \\ &\qquad + p_2 p_3 f_{\mathcal{D}_2}(x_1) f_{\mathcal{D}_3}(y - x_1) + p_3 p_2 f_{\mathcal{D}_3}(x_1) f_{\mathcal{D}_2}(y - x_1) \Bigg) dx_1, \end{aligned}$$

leading to

$$\begin{aligned} f_Y(y) = p_1^2 &\int_0^y f_{\mathcal{D}_1}(x_1) f_{\mathcal{D}_1}(y - x_1) dx_1 \\ &+ p_2^2 \int_0^y f_{\mathcal{D}_2}(x_1) f_{\mathcal{D}_2}(y - x_1) dx_1 + p_3^2 \int_0^y f_{\mathcal{D}_3}(x_1) f_{\mathcal{D}_3}(y - x_1) dx_1 \\ &+ p_1 p_2 \int_0^y f_{\mathcal{D}_1}(x_1) f_{\mathcal{D}_2}(y - x_1) dx_1 + p_2 p_1 \int_0^y f_{\mathcal{D}_2}(x_1) f_{\mathcal{D}_1}(y - x_1) dx_1 \\ &+ p_1 p_3 \int_0^y f_{\mathcal{D}_1}(x_1) f_{\mathcal{D}_3}(y - x_1) dx_1 + p_3 p_1 \int_0^y f_{\mathcal{D}_3}(x_1) f_{\mathcal{D}_1}(y - x_1) dx_1 \\ &+ p_2 p_3 \int_0^y f_{\mathcal{D}_2}(x_1) f_{\mathcal{D}_3}(y - x_1) dx_1 + p_3 p_2 \int_0^y f_{\mathcal{D}_3}(x_1) f_{\mathcal{D}_2}(y - x_1)) dx_1. \end{aligned}$$

Once more, we make use of the fact that $\int_0^y f_{\mathcal{D}_i}(x_1) f_{\mathcal{D}_j}(y - x_1) dx_1$ is the PDF of the sum $Z_1 + Z_2$ of two independent random variables, where $Z_1 \sim \mathcal{D}_i$ and $Z_2 \sim \mathcal{D}_j$ (now with $i, j \in \{1, 2, 3\}$). As before, we denote this density by $f_{i,j}$. Overall, we obtain

$$f_Y(y) = \sum_{i=1}^{3} \sum_{j=1}^{3} p_i p_j f_{i,j}(y),$$

or alternatively

$$f_Y(y) = \sum_{\ell_1=0}^{2} \sum_{\ell_2=0}^{2-\ell_1} \binom{2}{\ell_1} \binom{2-\ell_1}{\ell_2} p_1^{\ell_1} p_2^{\ell_2} p_3^{\ell_3} f_{(\ell_1, \ell_2, \ell_3)}(y), \tag{2}$$

where $\ell_1, \ell_2, \ell_3 = 2 - \ell_1 - \ell_2$ show how often cells of population 1, 2 and 3 are present in the pool. Again, $f_{(\ell_1, \ell_2, 2-\ell_1-\ell_2)}(y)$ is connected to $f_{i,j}$. For example, $f_{(0,1,1)}(y) = f_{2,3}(y)$ and $f_{(2,0,0)}(y) = f_{1,1}(y)$.

PDF of n-cell measurements of three populations (n arbitrary, $T = 3$)

Next, we suppose that we measure pools of n cells originating from three cell populations. Let $Y = X_1 + \ldots + X_n$. Then Equation (2) turns into

$$f_Y(y) = \sum_{\ell_1=0}^{n} \sum_{\ell_2=0}^{n-\ell_1} \binom{n}{\ell_1} \binom{n-\ell_1}{\ell_2} p_1^{\ell_1} p_2^{\ell_2} p_3^{\ell_3} f_{(\ell_1,\ell_2,\ell_3)}(y), \tag{3}$$

where $p_3 = 1 - p_1 - p_2$ and $\ell_3 = n - \ell_1 - \ell_2$.

PDF of n-cell measurements of T populations (n and T arbitrary)

Finally, we extend Equation (3) to the most general case, where n-cell pools are measured from a tissue that consists of T cell populations. Here, we obtain

$$f_Y(y) = \sum_{\ell_1=0}^{n} \sum_{\ell_2=0}^{n-\ell_1} \cdots \sum_{\ell_{T-1}=0}^{n-\ell_1-\ldots-\ell_{T-1}} \binom{n}{\ell_1} \binom{n-\ell_1}{\ell_2} \cdots \binom{n-\ell_1-\ldots-\ell_{T-2}}{\ell_{T-1}} p_1^{\ell_1} \cdots p_T^{\ell_T} f_{(\ell_1,\ldots,\ell_T)}(y),$$

where $p_T = 1 - p_1 - \ldots - p_{T-1}$ and $\ell_T = n - \ell_1 - \ldots - \ell_{T-1}$. The binomial coefficients form together the multinomial coefficient

$$\begin{aligned} &\binom{n}{\ell_1} \binom{n-\ell_1}{\ell_2} \cdots \binom{n-\ell_1-\ldots-\ell_{T-2}}{\ell_{T-1}} \\ &\qquad = \frac{n!(n-\ell_1)!\cdots(n-\ell_1-\ldots-\ell_{T-2})!}{\ell_1!\ell_2!\ldots\ell_{T-1}!(n-\ell_1)!(n-\ell_1-\ell_2)!\cdots(n-\ell_1-\ldots-\ell_{T-1})!} \\ &\qquad = \frac{n!}{\ell_1!\ell_2!\cdots\ell_T!} = \binom{n}{\ell_1,\ldots,\ell_T}. \end{aligned}$$

Taken together, this leads to the final PDF (3) in Section Small-pool models of heterogeneous gene expression:

$$f_Y(y) = \sum_{\ell_1=0}^{n} \sum_{\ell_2=0}^{n-\ell_1} \cdots \sum_{\ell_{T-1}=0}^{n-\ell_1-\ldots-\ell_{T-1}} \binom{n}{\ell_1,\ldots,\ell_T} p_1^{\ell_1} \cdots p_T^{\ell_T} f_{(\ell_1,\ldots,\ell_T)}(y).$$

The terms $\binom{n}{\ell_1,\ldots,\ell_T} p_1^{\ell_1} \cdots p_T^{\ell_T}$ are probabilities arising from the multinomial distribution and can be seen as multinomial weights of the densities $f_{(\ell_1,\ldots,\ell_T)}(y)$.
