# Supplementary material for: stochprofML: stochastic profiling using maximum likelihood estimation in R
Source: BMC Bioinformatics. 2021 Mar 15;22:123. doi: 10.1186/s12859-021-03970-7 (PMC7958472; doi:10.1186/s12859-021-03970-7)
Supplement: Supplementary file 2 — Additional file 2: PDF of pooled gene expression for mixed pool size vectors. Derivation of the PDF of samples that contain different cell numbers. [file 12859_2021_3970_MOESM2_ESM.pdf]

stochprofML: stochastic profiling using maximum likelihood estimation in R

Lisa Amrhein and Christiane Fuchs

Additional File 2
PDF of pooled gene expression for mixed pool size vectors

When estimating a gene expression model from data, one may want to verify whether the estimated model adequately describes the data. In Figure 11, we did this by comparing the estimated PDF to the histogram of the data and to the true PDF: The orange curve was known since we treated the case of synthetic data. For the blue curve, we first estimated the model parameters and then plugged these in into the general model PDF. In case of a uniform pool size across all measurements, this procedure is straightforward. For a vector of pool sizes, i. e. a mix of e. g. 1-cell, 2-cell and 10-cell data, the PDF (see e. g. Figure 2B) is less obvious. We calculate this function as follows:

- For each cell number contained in the n-vector, calculate the PDF of the respective pool size and plug in the parameter estimates.
- Calculate the weighted sum of these PDFs — weighted according to the times the respective pool size occurs in the n-vector.

The resulting PDF approximates the PDF of a sample where the observations are based on the pool sizes of the considered n-vector. While this PDF describes a mixture distribution with randomly drawn pool sizes (according to the weights used), we in our applications assume the pool sizes to be known for each measurement.

```
mix.d.sum.of.mixtures.LNLN <- function(y, n.vector, p.vector, mu.vector,
+    sigma.vector){
+        densmix <- matrix(0, ncol = length(y), nrow = length(n.vector))
+        for(i in 1:length(n.vector)){
+            densmix[i, ] <- d.sum.of.mixtures.LNLN(y = y, n = n.vector[i],
+                p.vector = p.vector, mu.vector = mu.vector,
+                sigma.vector = sigma.vector, logdens = FALSE)
+        }
+        Dens<-colSums(densmix)/length(n.vector)
+    }
```
