# Supplementary material for: stochprofML: stochastic profiling using maximum likelihood estimation in R
Source: BMC Bioinformatics. 2021 Mar 15;22:123. doi: 10.1186/s12859-021-03970-7 (PMC7958472; doi:10.1186/s12859-021-03970-7)
Supplement: Supplementary file 3 — Additional file 3: Transformation of population probabilities. Details about the transformation of the population probabilities during parameter optimization. [file 12859_2021_3970_MOESM3_ESM.pdf]

stochprofML: stochastic profiling using maximum likelihood estimation in R

Lisa Amrhein and Christiane Fuchs

Additional File 3
Transformation of population probabilities

As described in the main paper in the Implementation section, we transform the model parameters before optimization of the likelihood function such that no constraints of the parameter space have to be accounted for. Here, we provide details about the transformation of the population probabilities.

In case of two populations, there is only one parameter $p \in [0,1]$ that determines the probabilities p and $1-p$ of populations 1 and 2. We transform p to

$$w = \text{logit}(p) = \log\left(\frac{p}{1-p}\right) \in \mathbb{R}$$

and later back-transform this via

$$p = \text{logit}^{-1}(w) = \text{expit}(w) = \frac{\exp(w)}{1+\exp(w)} \in [0,1] \; .$$

The advantage of w as compared to p is the unrestricted range $\mathbb{R}$ instead of $[0,1]$. In case of $T > 2$ populations, the probabilities $p_1, \ldots, p_T$ have to fulfill $p_h \in [0,1]$ for all $h = 1, \ldots, T$ and $\sum_{h=1}^{T} p_h = 1$. We set $\tilde{p}_h = p_1 + \cdots + p_h$ and use the following transformations

$$w_h = \text{logit}\left(\frac{p_1 + \cdots + p_h}{p_1 + \cdots + p_{h+1}}\right) = \text{logit}\left(\frac{\tilde{p}_h}{\tilde{p}_{h+1}}\right) \in \mathbb{R} \qquad \text{for all } h \in 1, \ldots, T-1.$$

For the back-transformations, we start at $h = T-1$ and calculate

$$\tilde{p}_h = \text{expit}(w_h)\, \tilde{p}_{h+1} \in [0,1] \qquad \text{for all } h \in T-1, \ldots, 1$$

in reverse order. We set $\tilde{p}_T = 1$ to ensure that the probabilities sum up to one. Additionally, one has $\tilde{p}_h \leq \tilde{p}_{h+1}$ as $\text{expit}(w_h) \in [0,1]$ for all $h \in 1, \ldots, T-1$. Obviously, $p_1 = \tilde{p}_1$, and the remaining population probabilities are given by

$$p_h = \tilde{p}_h - \tilde{p}_{h-1} \in [0,1] \qquad \text{for all } h \in 2, \ldots, T.$$

The (back-)transformations are implemented in `transform.par()` and `backtransform.par()`.
