# Supplementary material for: stochprofML: stochastic profiling using maximum likelihood estimation in R
Source: BMC Bioinformatics. 2021 Mar 15;22:123. doi: 10.1186/s12859-021-03970-7 (PMC7958472; doi:10.1186/s12859-021-03970-7)
Supplement: Supplementary file 4 — Additional file 4: Derivation of sample composition probabilities. Derivation of the conditional probability of a cell composition given the measured gene expression needed in Section Prediction of sample compositions. [file 12859_2021_3970_MOESM4_ESM.pdf]

stochprofML: stochastic profiling using maximum likelihood estimation in R

Lisa Amrhein and Christiane Fuchs

Additional File 4
Derivation of sample composition probabilities

We describe how to predict the population composition of a cell pool, as applied in Section Prediction of sample compositions. A key formula here is the conditional probability of a cell composition given the measured gene expression, which we derive here. We use the following notations and assumptions:

- The overall gene expression of a cell pool is denoted by Y and assumed a continuous random variable with PDF $f_Y(y)$.
- $L = (L_1, \ldots, L_T)$ denotes the specific cell population combinations, i. e. L_i is the number of cells of population i for all $i = 1, \ldots, T$, within a pool of $L_1 + \ldots + L_T$ cells. L is a discrete random vector with PMF $P(L = \ell)$.
- $f_{Y|L=\ell}(y)$ is the conditional PDF of the overall gene expression in a cell pool whose composition is known to equal ℓ. For shorter notation, this was referred to as $f_{(\ell_1,\ell_2,\ldots,\ell_T)}(y_i|\boldsymbol{\theta})$ in Section Small-pool models of heterogeneous gene expression.
- In turn, $P(L = \ell|Y = y)$ is the conditional PMF of the cell pool composition given the pool gene expression measurement $Y = y$.

We use Bayes' theorem to derive the latter PMF:

$$P(L = \ell|Y = y) = \frac{f_{Y|L=\ell}(y)P(L = \ell)}{f_Y(y)} = \frac{f_{Y|L=\ell}(y)P(L = \ell)}{\sum_{j\in J} f_{Y|L=j}(y)P(L = j)}, \qquad (1)$$

where J is the set of all possible compositions of the cell pool, i. e. the set of all vectors $(j_1, \ldots, j_T)$ with $j_i \in \mathbb{N}_0$ and $j_1 + \ldots + j_T = \ell_1 + \ldots + \ell_T$.

The terms in Equation (1) depend on the population probabilities $\boldsymbol{p} = (p_1, \ldots, p_T)$ and the gene expression model (in this work: LN-LN, rLN-LN, or EXP-LN), characterized by its respective parameters. We assume the expression model to be fixed and denote all model parameters (including $\boldsymbol{p}$) by $\boldsymbol{\theta}$. In practice, $\boldsymbol{\theta}$ is unknown, and hence we use its maximum likelihood estimates here.

Given the estimate $\hat{\boldsymbol{p}}$ of $\boldsymbol{p}$, $L = \ell = (\ell_1, \ldots, \ell_T)$ approximately follows a multinomial distribution with parameters $n = \ell_1 + \ldots + \ell_T$ and $\hat{\boldsymbol{p}}$. The PMF of the cell pool composition $(\ell_1, \ldots, \ell_T)$ hence reads

$$P(L = (\ell_1, \ldots, \ell_T)) = \binom{n}{\ell_1, \ell_2, \ldots, \ell_T} \hat{p}_1^{\ell_1} \hat{p}_2^{\ell_2} \cdots \hat{p}_T^{\ell_T},$$

where $\binom{n}{\ell_1,\ell_2,\ldots,\ell_T} = \frac{n!}{\ell_1!\,\ell_2!\cdots\ell_T!}$ is the multinomial coefficient. With this, the conditional PMF of the cell pool composition given the pooled gene expression measure-

ment Y reads:

$$P(L=\ell|Y=y)=\frac{f_{Y|L=\ell}(y;\hat{\boldsymbol{\theta}})\binom{n}{\ell_1,\ell_2,\ldots,\ell_T}\hat{p}_1^{\ell_1}\hat{p}_2^{\ell_2}\cdots\hat{p}_T^{\ell_T}}{f_Y(y;\hat{\boldsymbol{\theta}})} \tag{2}$$

$$=\frac{f_{Y|L=\ell}(y;\hat{\boldsymbol{\theta}})\binom{n}{\ell_1,\ell_2,\ldots,\ell_T}\hat{p}_1^{\ell_1}\hat{p}_2^{\ell_2}\cdots\hat{p}_T^{\ell_T}}{\sum_{j\in J}f_{Y|L=j}(y;\hat{\boldsymbol{\theta}})\binom{n}{j_1,j_2,\ldots,j_T}\hat{p}_1^{j_1}\hat{p}_2^{j_2}\cdots\hat{p}_T^{j_T}}.$$
