# Supplementary material for: stochprofML: stochastic profiling using maximum likelihood estimation in R
Source: BMC Bioinformatics. 2021 Mar 15;22:123. doi: 10.1186/s12859-021-03970-7 (PMC7958472; doi:10.1186/s12859-021-03970-7)
Supplement: Supplementary file 5 — Additional file 5: Interactive Functions. Examples how the interactive usage of stochprofML works. [file 12859_2021_3970_MOESM5_ESM.pdf]

stochprofML: stochastic profiling using maximum likelihood estimation in R

Lisa Amrhein and Christiane Fuchs

Additional File 5
Interactive Functions

As indicated in Section Usage of stochprofML, we show an example of the interactive functions for data generation, `stochasticProfilingData()`, and parameter estimation, `stochasticProfilingML()`.

Synthetic data generation: `stochasticProfilingData()`

```
R> library("stochprofML")
R> set.seed(10)
R> stochprofML::stochasticProfilingData()
This function generates synthetic data from the stochastic profiling model.
In the following, you are asked to specify all settings. By pressing 'enter',
you choose the default option.

---------
Please choose the model you would like to generate data from:
1: LN-LN
2: rLN-LN
3: EXP-LN
(default: 1)
R> 1
---------
Please enter the number of different populations you would like to consider:
(default: 2)
R> 2
---------
Please enter the number of stochastic profiling observations you wish to generate:
(default: 100)
R> 1000
---------
Next we enter the number of cells that should enter each observation,
which case do you want:
1: all observations should contain the same number of cells, or
2: each observation contains a different number of cells
(default: 1).
R> 1
---------
Please enter the number of cells that should enter each sample:
(default: 10)
```

```
R> 10

---------
Please enter the number of co-expressed genes you would like to collect
in one cluster
(default: 1)

R> 1

---------
Please enter the probabilities for each of the 2 populations, e.g. type
0.62, 0.38
or
0.62 0.38.
It is recommended to choose the order of the populations such that
(for the first gene, if there is more than one)
log-mean for population 1 >= log-mean for population 2 >= ...

R> 0.62, 0.38

---------
Please enter the log-means for each of the 2 populations, e.g. type
0.47, -0.87.

R> 0.47, -0.87

---------
Please enter the log-standard deviation, which is the same for all
populations, i.e. type e.g.
0.03
irrespectively of the number of populations.

R> 0.03

---------
Would you like to write the generated dataset to a file? (Be careful not to
overwrite any existing file!) Please type 'yes' or 'no'.

R> yes

Please enter a valid path and filename, either a full path, e.g.
D:/Users/lisa.amrhein/Desktop/mydata.txt
or just a file name, e.g.
mydata.txt.
The current directory is
D:/Users/lisa.amrhein/Desktop.

test.txt

Hit <Return> to see next plot:

R> <Return>
```

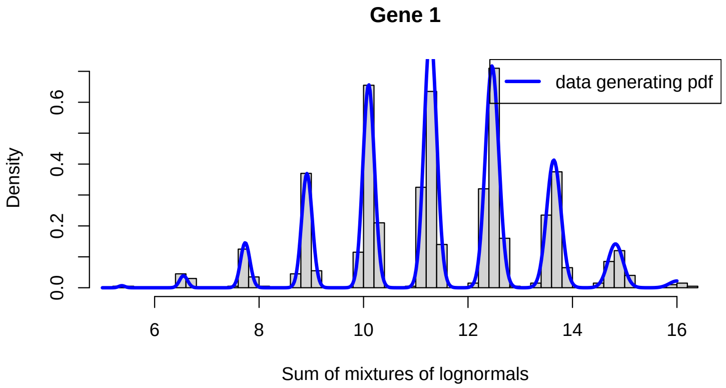


```
The dataset has been generated. The first 50 observations are:

gene 1
observation 1  12.444051
observation 2  12.406390
observation 3  12.412508
observation 4  11.274005
observation 5  12.455432
observation 6  11.255553
observation 7  11.281351
observation 8  13.587420
observation 9  11.222902
observation 10 12.586923
observation 11 12.278757
observation 12 14.746502
observation 13 12.313032
observation 14 12.387004
observation 15 10.007876
observation 16  9.978140
observation 17 11.461335
observation 18 10.015642
observation 19 12.575700
observation 20 12.411795
observation 21  9.965103
observation 22 10.139115
observation 23 12.437228
observation 24 10.039271
observation 25 12.489048
observation 26 11.218177
observation 27 13.217671
observation 28 13.683550
observation 29  8.877630
observation 30 12.550239
observation 31  9.922716
```

```
observation 32 10.110123
observation 33  8.720011
observation 34 10.237112
observation 35 11.223407
observation 36 12.468208
observation 37 12.561782
observation 38 15.013314
observation 39 10.151065
observation 40 12.405660
observation 41 13.490263
observation 42 14.675084
observation 43 11.432289
observation 44 13.565312
observation 45 10.076739
observation 46 12.414651
observation 47  8.861460
observation 48 12.401696
observation 49 11.271324
observation 50 13.750237


The full dataset has been written to
test.txt.
It is also stored in the .Last.value variable.
```

Parameter estimation: `stochasticProfilingML()`

```
R> library("stochprofML")
R> set.seed(20)
R> stochprofML::stochasticProfilingML()
This function performs maximum likelihood estimation for the stochastic
profiling model. In the following, you are asked to enter your data and
specify some settings. By pressing 'enter', you choose the default option.

---------
How would you like to input your data?
1: enter manually
2: read from file
3: enter the name of a variable
(default: 1).
R> 2
---------
The file should contain a data matrix with one dimension standing for genes
and the other one for observations. Fields have to be separated by tabs or white
spaces, but not by commas. If necessary, please delete the commas in the
text file using the 'replace all' function of your text editor.
```

```
Please enter a valid path and filename, either a full path, e.g.
D:/Users/lisa.amrhein/Desktop/mydata.txt
or just a file name, e.g.
mydata.txt.
The current directory is
D:/Users/lisa.amrhein/Desktop.
R> test.txt
Does the file contain column names? Please enter 'yes' or 'no'.
R> yes
Does the file contain row names? Please enter 'yes' or 'no'.
R> no
Do the columns stand for different genes or different observations?
1: genes
2: observations.
R> 1
This is the head of the dataset (columns contain different genes):
gene.1
[1,] 12.44405
[2,] 12.40639
[3,] 12.41251
[4,] 11.27400
[5,] 12.45543
[6,] 11.25555


If the matrix does not look correct to you, there must have been an error
in the answers above. In this case, please quit by pressing 'escape' and call
stochasticProfilingML() again.

The file contained the following gene names:
gene.1
R> no
---------
Please choose the model you would like to estimate:
1: LN-LN
2: rLN-LN
3: EXP-LN
(default: 1)
R> 1
---------
Please enter the number of different populations you would like to estimate:
(default: 2)
R> 2
---------
Please enter the number of cells that entered each observation, either
1: all observations contain the same number of cells, or
```

```
2: each observation contains a different number of cells
(default: 1).
R> 1
---------
Please enter the number of cells that should enter each observation:
(default: 10)
R> 10

***** Estimation started! *****



Maximum likelihood estimate (MLE):
p_1 mu_1_gene_gene.1 mu_2_gene_gene.1            sigma
0.6142           0.4690          -0.8650           0.0290

Value of negative log-likelihood function at MLE:
1124.932

Violation of constraints:
none

BIC:
2277.496

Approx. 95% confidence intervals for MLE:
lower       upper
p_1               0.60461644  0.62369584
mu_1_gene_gene.1  0.46779634  0.47020366
mu_2_gene_gene.1 -0.87085629 -0.85914371
sigma             0.02774407  0.03031279

Top parameter combinations:
p_1 mu_1_gene_gene.1 mu_2_gene_gene.1 sigma   target
[1,] 0.6142           0.469          -0.865 0.029 1124.932
[2,] 0.6142           0.469          -0.865 0.029 1124.932
[3,] 0.6141           0.469          -0.865 0.029 1124.932
[4,] 0.6142           0.469          -0.865 0.029 1124.932
[5,] 0.6142           0.469          -0.865 0.029 1124.933
[6,] 0.6142           0.469          -0.865 0.029 1124.933
```
