# Supplementary material for: stochprofML: stochastic profiling using maximum likelihood estimation in R
Source: BMC Bioinformatics. 2021 Mar 15;22:123. doi: 10.1186/s12859-021-03970-7 (PMC7958472; doi:10.1186/s12859-021-03970-7)
Supplement: Supplementary file 6 — Additional file 6: Details on Simulation Studies. More information and further details on the Simulation study on optimal pool size, on the Simulation study on impact of parameter values and on the Simulation study on the uncertainty of pool sizes. [file 12859_2021_3970_MOESM6_ESM.pdf]

# stochprofML: Stochastic Profiling Using Maximum Likelihood Estimation in R

Lisa Amrhein and Christiane Fuchs

## Additional File 6
## Details on Simulation Studies

The general procedure in the simulation studies in the Results and Discussion part of the main text is to first generate synthetic datasets with some predefined population parameters and frequencies using `r.sum.of.mixtures()`. Thereby datasets with either fixed or varying pool sizes are generated, i. e. the numbers of cells contained in one pool are fixed or vary from cell pool to cell pool within a dataset. Next, we assume that we do not know the predefined model parameters and estimate them using `stochprof.loop()`. Then we compare the estimates of the parameters in different ways, e. g. how they are influenced by increasing cell numbers or how their variance differs when the dataset was generated with differing population parameters.

Here, we give an overview about the different model parameter settings and pool sizes used in data generation: We use datasets with fixed pool sizes that contain single-cells, 2 cells, 5 cells, 10 cells, 15 cells, 20 cells or 50 cells. Additionally, we chose two types of datasets with varying pool sizes. The first contains small cell pools with 1, 2, 5 and 10 cells, the second contains larger cell pools with 10, 15, 20 and 50 cells. Thus, in total we have nine different cell pool settings that we use for data generation.

In all simulation studies, we use the LN-LN model with five different parameter settings, given in Table S1. While the first set is considered to be the default, each of the other parameter sets differs from it in one of the population parameters. Taken together, for each of the nine cell pool settings and each of the five parameter

| | $p$ | $\mu_1$ | $\mu_2$ | $\sigma$ |
|---|---|---|---|---|
| Set 1 | 0.2 | 2 | 0 | 0.2 |
| Set 2 | 0.1 | 2 | 0 | 0.2 |
| Set 3 | 0.4 | 2 | 0 | 0.2 |
| Set 4 | 0.2 | 2 | 1 | 0.2 |
| Set 5 | 0.2 | 2 | 0 | 0.5 |

**Table S1 Overview of the five model parameter settings used in the Simulation study on optimal pool size and in the Simulation study on impact of parameter values.**

settings 1,000 datasets are generated using `r.sum.of.mixtures.LNLN()`, so that in total we have `5*9*1000` $= 4.5 \times 10^4$ simulated datasets.

### Impact of pool sizes

In the first simulation study (Section Simulation study on optimal pool size), we investigate how parameter estimation is influenced by increasing cell numbers within

the cell pools. The results for parameter set 1 are displayed in the main part of the manuscript. Here, we show the corresponding results for the remaining four parameter settings.

In the second parameter setting, the fraction of the first population was reduced to 10% as compared to the first parameter setting. The results are shown in Figure S1. They are similar to the results of the first parameter set in Figure 6. For set 2, however, single cells lead to large variance of estimates, supposedly due to the small sample size of 50 in combination with the small probability (10%) of the first population: We can only expect five single cells of the first population to be measured on average. In some datasets, this will be too low to estimate the parameters of the first population and/or their proportion satisfactorily. Consequently, the violins of the single-cell estimates show a higher variance, especially for the estimates of the parameters of the first population. In the third parameter setting, the fraction

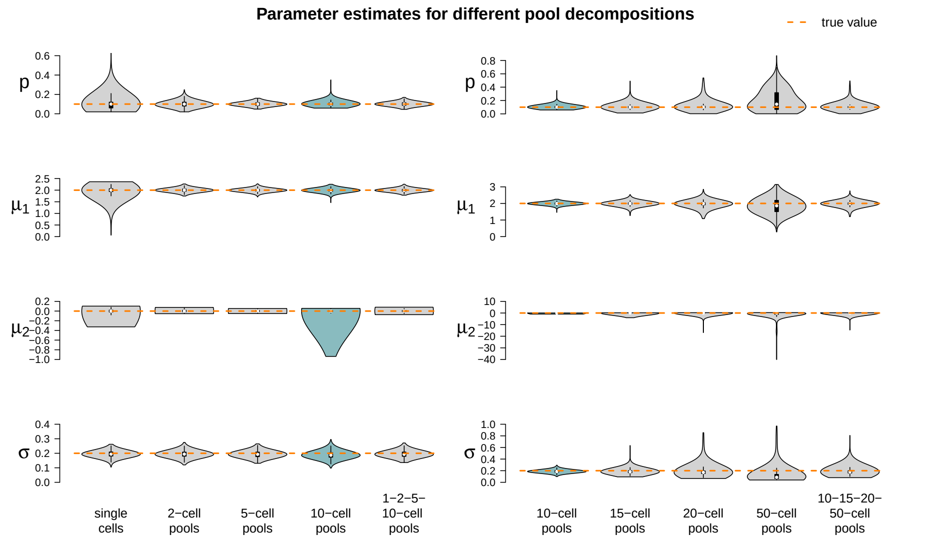


**Figure S1** Estimated parameters of LN-LN-model on 9,000 simulated datasets, i. e. 1,000 datasets of each pool composition generated with parameter set 2 (see Table S1). *Left:* Accumulated parameter fits of the single-cell, 2-cell, 5-cell, 10-cell and mixture of single-, 2-, 5- and 10-cell pools. *Right:* Results of the 10-cell pools are repeated (turquoise violins), next to those of the larger pool sizes, namely 15-, 20-, 50-cells and their mixture. Each violin is based on 1,000 parameter estimates. The true parameter values are marked in orange.

of the first population was increased to 40%. The resulting estimates are shown in Figure S2. In this setting, both populations are similarly frequent; hence, it seems plausible that the single-cell estimates show similar variability as for example the 2-cell estimates. The estimates of the mixed pools of the lower cell numbers provide estimates that are as accurate as the ones for single-cell and 2-cell data. From a pool size of five cells on, the estimates vary strongly. Apparently, low cell numbers are advisable if a tissue is not dominated by one cell population. In the fourth parameter setting, $\mu_2$ is increased to 1 and thus larger than in the first parameter setting. The two populations are more similar. The resulting estimates are shown in Figure S3. Starting from a pool size of 10 cells, it seems as if the variance of the estimates did not increase any more. The estimates for the mixed pools with larger cell numbers can sometimes not distinguish the populations, therefore the

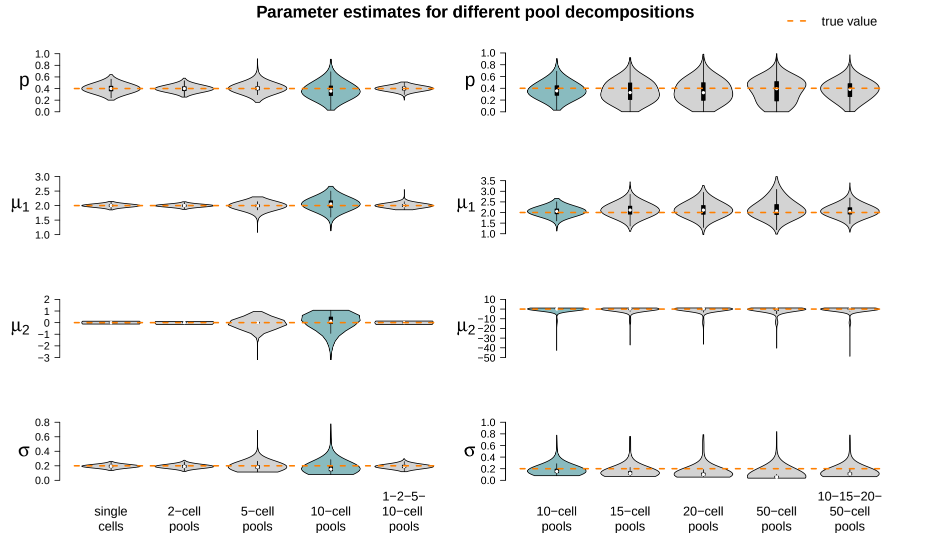


**Figure S2** Parameter estimates as in Figure S1 but for parameter set 3 (see Table S1).

violin of $p$ is bi-modal. We draw the same conclusion as for two populations with similar frequencies that more similar populations should be investigated in pools with lower cell numbers because their individual expression profile is blurred for small pool sizes already. Finally, we investigate the effect of different pool sizes in

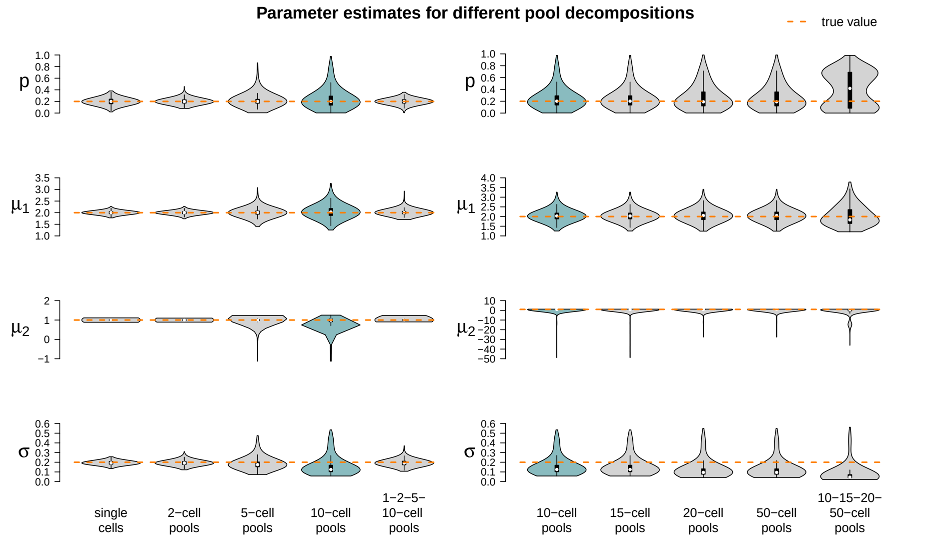


**Figure S3** Parameter estimates as in Figure S1 but for parameter set 4 (see Table S1).

the fifth parameter set, where the log-sd $\sigma$ of both populations is increased to 0.5. The resulting estimates of the model parameters are shown in Figure S4. With an increase of $\sigma$, both populations have broader distributions. It appears that there is an increase in variance in the estimates between the 5-cell and the 10-cell measurements. Increasing cell numbers in the pools mainly influences the estimate of $\sigma$, which is increasingly underestimated.

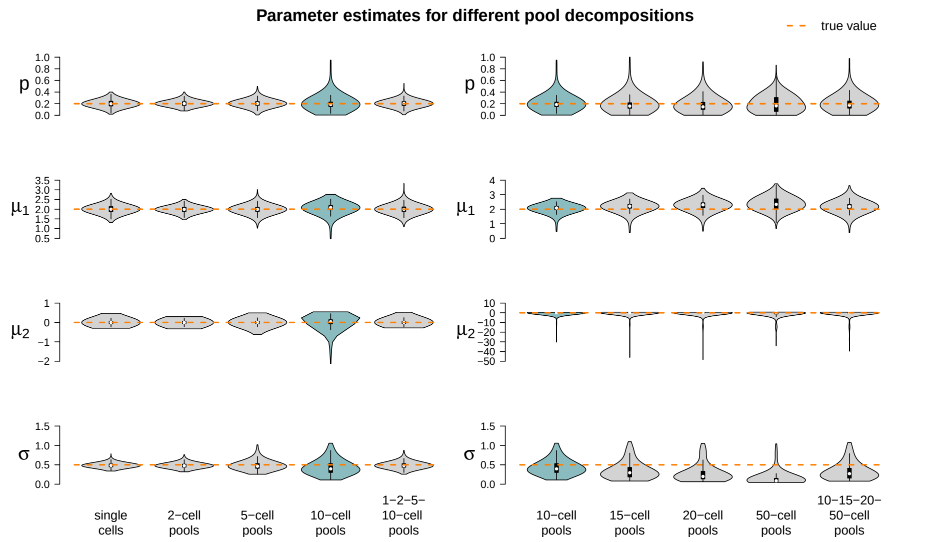


**Figure S4** Parameter estimates as in Figure S1 but for parameter set 5 (see Table S1).

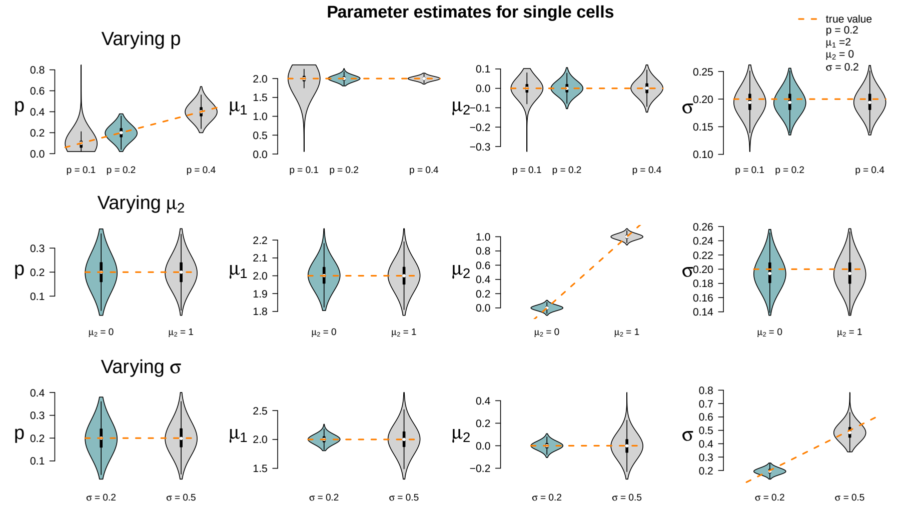


**Figure S5** Parameter estimates for single-cell data and varying parameter values: Synthetic data is generated using the LN-LN model for varying values of $p$, $\mu_2$ and $\sigma$. Results for the standard setting $p = 0.2$, $\mu_1 = 2$, $\mu_2 = 0$ and $\sigma = 0.2$ are shown in turquoise, results for four more settings in grey. For each setting, we generate 1,000 synthetic datasets and back-infer the model parameters. Violin plots summarize the 1,000 estimates. The underlying true parameter values are marked in orange.

## Impact of parameter values

In Section Simulation study on impact of parameter values, we investigate the influence of the model parameter values on the estimation performance while fixing the pool size. In the main part of the manuscript, we presented results for 10-cell pools (see Figure 7). Here, corresponding analyses for the remaining eight cell pool sizes ($n \in \{1, 2, 5, 15, 20, 50\}$ and two mixtures) are shown.

Results for single-cell and 2-cell pools look alike (Figures S5 and S6). As discussed before, the variance of the estimates become large for a small value of $p$ in combination with the small pool sizes. For both single-cell and 2-cell data, varying $\mu_2$ does not affect the estimation accuracy of the estimation, whereas a larger value of $\sigma$ leads to higher variance of all parameter estimates but for $p$. In contrast to this, the 5-cell data results in a different pattern (Figure S7): As compared to the estimates from the standard setting, the estimates show a larger variance. The mixture of small cell pool numbers (Figure S8), however, lead to similar results as the pure 2-cell datasets. Figure S9 displays the results for the 15-cell data. For most parameter combinations, the variance of the estimates does not change dramatically. The most accurate estimates are achieved for small $p$, the least accurate ones for large $\sigma$, in which case $\sigma$ gets underestimated. The same holds true for the 20- and 50-cell datasets (Figures S10 and S11), with even larger variance. For the mixture of large cell pools (Figure S12), estimation performance is comparable to the one for the pure 50-cell measurements.

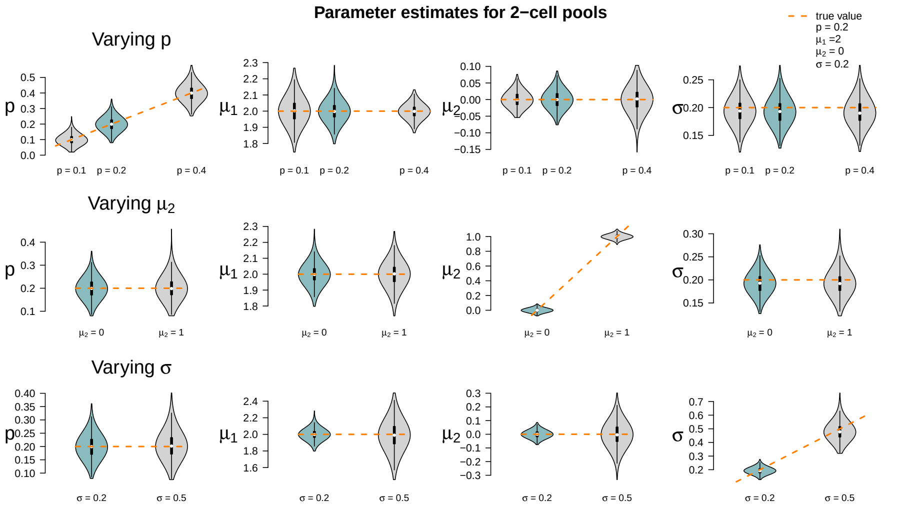


**Figure S6** As Figure S5, but for 2-cell data.

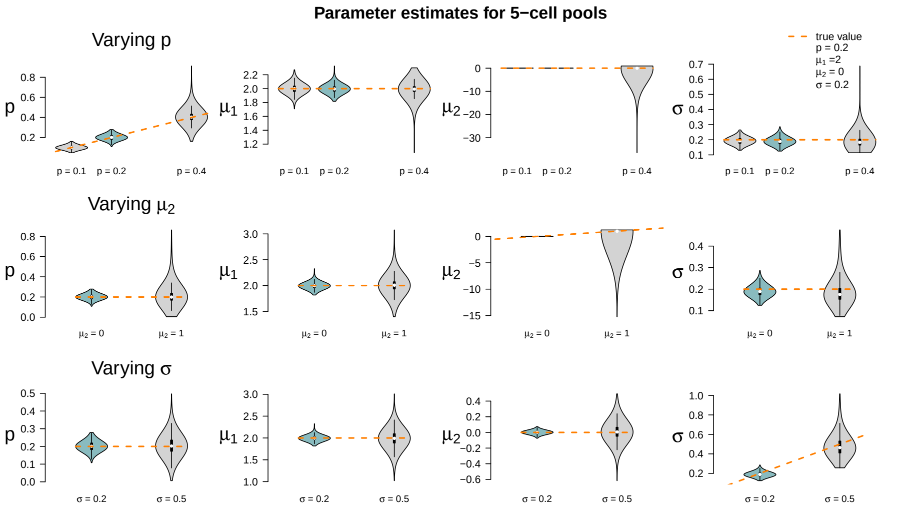


**Figure S7** As Figure S5, but for 5-cell data.

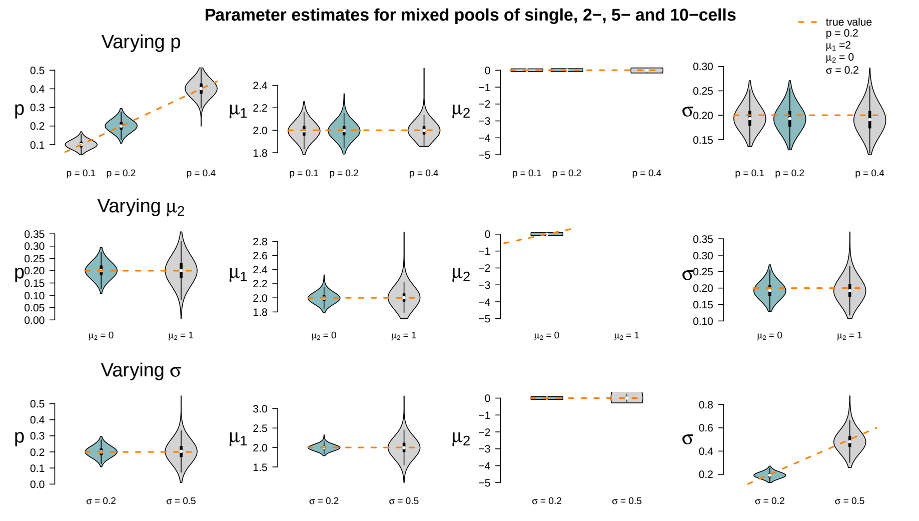


**Figure S8** As Figure S5, but for a mixture of single-, 2-, 5- and 10-cell data.

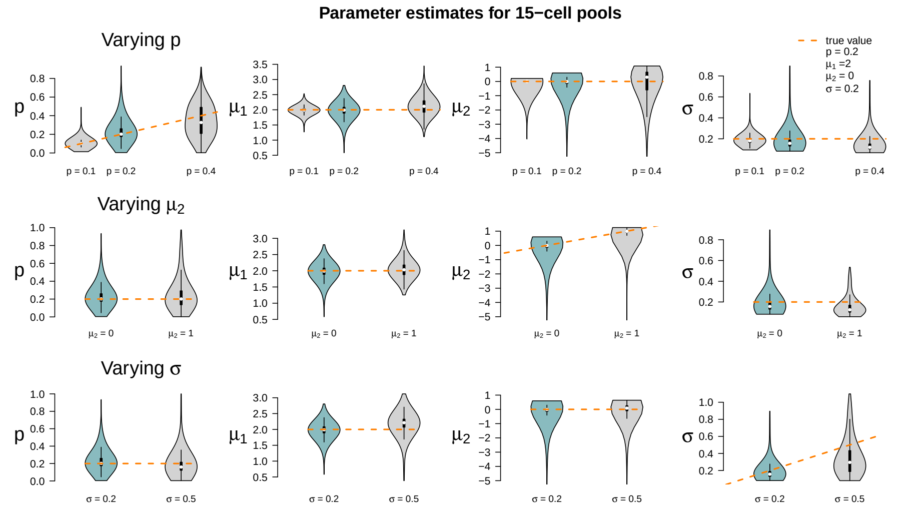


**Figure S9** As Figure S5, but for 15-cell data.

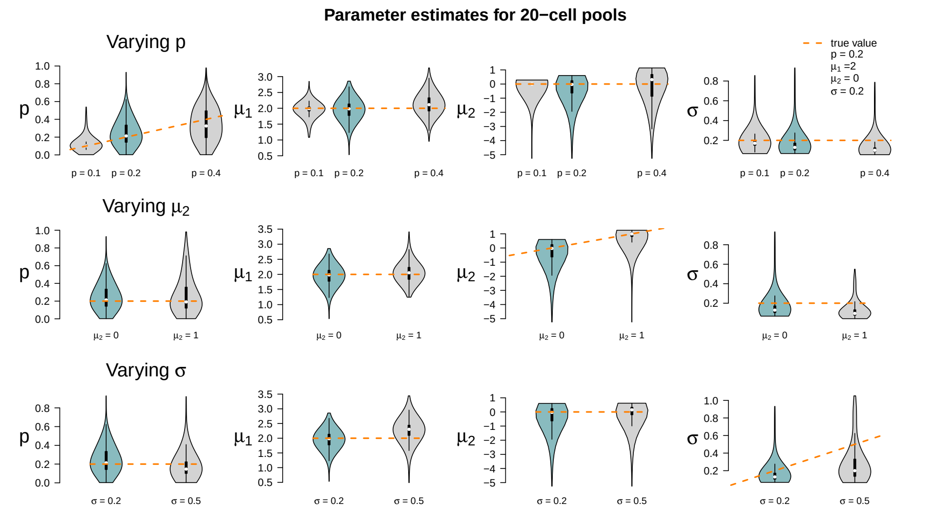


**Figure S10** As Figure S5, but for 20-cell data.

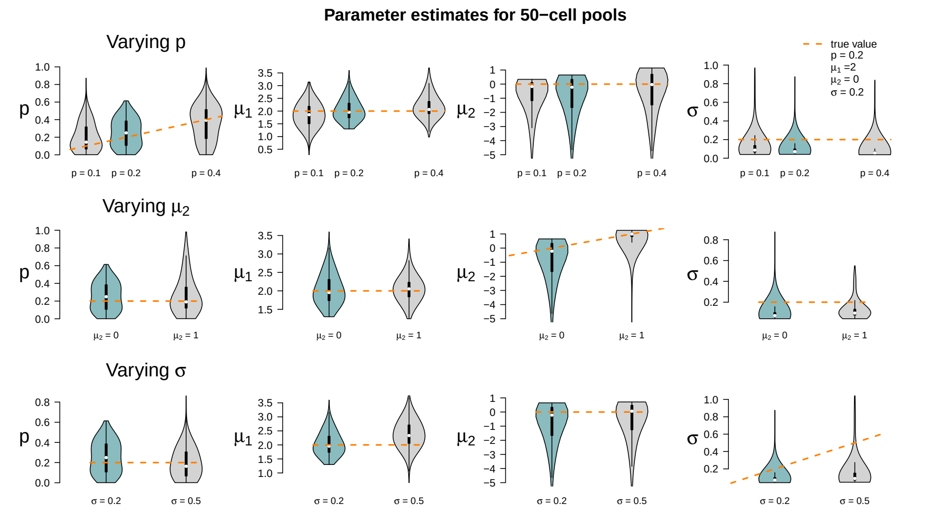


**Figure S11** As Figure S5, but for 50-cell data.

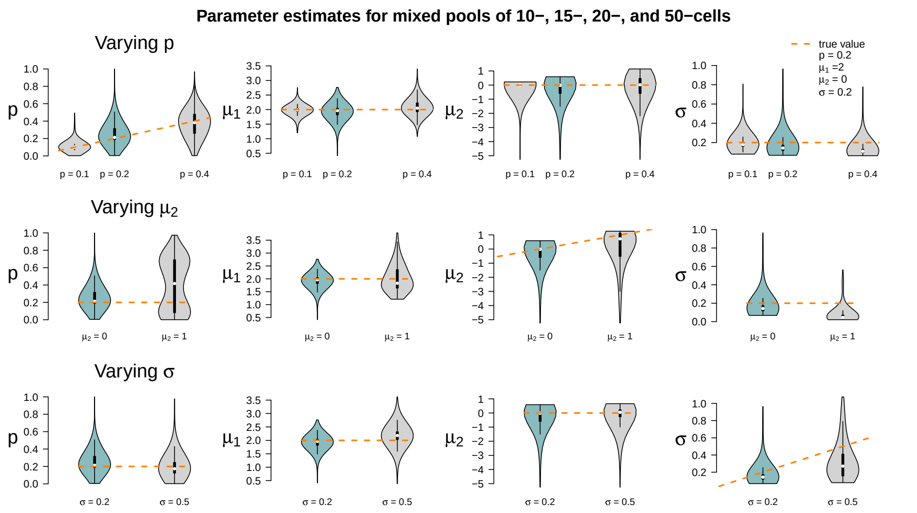


**Figure S12** As Figure S5, but for a mixture of 10-, 15-, 20- and 50-cell data.
